# Refermentation and maturation of lambic beer in bottles: a necessary step for gueuze production

**DOI:** 10.1128/aem.01869-23

**Published:** 2024-03-06

**Authors:** Dries Bongaerts, Arne Bouchez, Jonas De Roos, Margo Cnockaert, Anneleen D. Wieme, Peter Vandamme, Stefan Weckx, Luc De Vuyst

**Affiliations:** 1 Department of Bioengineering Sciences, Research Group of Industrial Microbiology and Food Biotechnology (IMDO), Faculty of Sciences and Bioengineering Sciences, Vrije Universiteit Brussel, Brussels, Belgium; 2 Department of Biochemistry and Microbiology, Laboratory for Microbiology, Faculty of Sciences, Ghent University, Ghent, Belgium; 3 Department of Biochemistry and Microbiology, BCCM/LMG Bacteria Collection, Faculty of Sciences, Ghent University, Ghent, Belgium; INRS Armand-Frappier Sante Biotechnologie Research Centre, Laval, Canada

**Keywords:** lambic beer, gueuze, lactic acid bacteria, acetic acid bacteria, *Brettanomyces*, MALDI-TOF MS, metagenetics, metabolomics

## Abstract

The production of gueuze beers through refermentation and maturation of blends of lambic beer in bottles is a way for lambic brewers to cope with the variability among different lambic beer batches. The resulting gueuze beers are more carbonated than lambic beers and are supposed to possess a unique flavor profile that varies over time. To map this refermentation and maturation process for gueuze production, a blend of lambic beers was made and bottled, whereby one of them was produced with the old wheat landrace *Zeeuwse Witte*. Through the use of matrix-assisted laser desorption/ionization time-of-flight mass spectrometry and high-throughput sequencing of bacterial and fungal amplicons, in combination with metabolite target analysis, new insights into gueuze production were obtained. During the initial stages of refermentation, the conditions in the bottles were similar to those encountered during the maturation phase of lambic beer productions in wooden barrels, which was also reflected microbiologically (presence of *Brettanomyces* species, *Pediococcus damnosus*, and *Acetobacter lambici*) and biochemically (ethanol, higher alcohols, lactic acid, acetic acid, volatile phenolic compounds, and ethyl esters). However, after a few weeks of maturation, a switch from a favorable environment to one with nutrient and dissolved oxygen depletion resulted in several changes. Concerning the microbiology, a sequential prevalence of three lactic acid bacterial species occurred, namely, *P. damnosus*, *Lentilactobacillus buchneri*, and *Lactobacillus acetotolerans*, while the diversity of the yeasts decreased. Concerning the metabolites produced, mainly those of the *Brettanomyces* yeasts determined the metabolic profiles encountered during later stages of the gueuze production.

**IMPORTANCE:**

Gueuze beers are the result of a refermentation and maturation process of a blend of lambic beers carried out in bottles. These gueuze beers are known to have a long shelf life, and their quality typically varies over time. However, knowledge about gueuze production in bottles is scarce. The present study provided more insights into the varying microbial and metabolite composition of gueuze beers during the first 2 years of this refermentation and maturation process. This will allow gueuze producers to gain more information about the influence of the refermentation and maturation time on their beers. These insights can also be used by gueuze producers to better inform their customers about the quality of young and old gueuze beers.

## INTRODUCTION

Belgian lambic beers are low-carbonized, low-alcohol sour beers, which are produced through spontaneous fermentation and maturation in horizontal wooden barrels (1–4). These beers can be enjoyed as such, but they usually serve as a base for the production of lambic-derived beers. One of these beers is gueuze, a blend of young lambic beer (1 year of maturation) and old lambic beer (usually 2 and 3 years of maturation), that undergoes an additional refermentation and maturation step in glass bottles placed horizontally in a cellar (1, 3, 5). This results in a non-bitter, acidic, fruity beer that is more carbonated and higher in alcohol content compared with the lambic beers started from (6–10).

The application of refermentation and maturation in bottles is often encountered for the production of alcoholic beverages (11). One of the most known examples is the production of sparkling wines (12). During bottling, sucrose and maturation yeast are added, resulting in a refermentation of the wine in the bottle that makes the wine sparkling. Refermentation in bottles is also used for the production of beers, more specifically ales, which have a higher alcohol content than lagers (13). After primary fermentation and maturation by *Saccharomyces cerevisiae* in stainless steel tanks, the beer is transferred to bottles, to which sugar and a maturation yeast strain of *S. cerevisiae* is added to perform a second fermentation and maturation step (11, 14, 15). Traditionally, refermentation of beers in bottles has been done to increase the carbonation level (14, 15). During this second fermentation step, carbon dioxide gas is produced through the yeast metabolism, improving foam formation and giving the beer a more refreshing taste. Later, the focus of beer refermentation in bottles has shifted toward an enhanced production of flavor compounds, such as higher alcohols and esters, which increases the fruitiness of the beer (14–18). Furthermore, refermentation of beer in bottles increases its shelf life because the yeast can use a part of the oxygen that is still present in the bottle. This delays the oxidation of the beer, which can otherwise cause a stale, cardboard flavor due to the formation of volatile aldehydes, more specifically 2-nonenal (18). Finally, also additional ethanol is produced during the refermentation process in bottles, which increases not only the alcohol content of the beer but also its microbial stability (19, 20).

For gueuze production, refermentation of the lambic beer blend also serves as a way to deal with batch differences that lambic brewers and blenders encounter across different barrels because of the spontaneous character of lambic beer production (https://www.bjcp.org/style/2015/23/23D/lambic/). Blending of lambic beers, which is mainly based on the experience and taste assessment of the brewer, makes the production of a more uniform end-product possible, although the resulting gueuze beer still remains variable as such. Traditional gueuze production proceeds through further spontaneous fermentation and maturation of the sugar added. However, some gueuze beers are produced by the addition of sugar and a starter culture consisting of a pure yeast culture to accelerate the refermentation of the blend in the bottles, mainly from a commercial point of view (21). There are even gueuze producers that do not apply a refermentation step in bottles. In this case, the (young) lambic beer is mixed with beer (lambic beer and even ales), filtered, carbonized, pasteurized, and bottled. Sometimes, sugar is added to sweeten the beer. To make a distinction between these commercial versions of gueuze beers and the more traditional ones, the term *oude geuze* (old gueuze) was introduced, which is legally protected at the European level (22). To produce *oude geuze*, the weighted average age of the lambic beers used should be at least 1 year, and part of the lambic beer had to be aged at least 3 years in wooden barrels. Furthermore, a refermentation process in bottles is required. Additional requirements are a maximal concentration of 0.5 ppm of isoamyl acetate, a minimal concentration of 50 ppm of ethyl acetate, a minimal volatile acidity of 10 meq of NaOH, and a minimal total acidity of 75 meq of NaOH.

Limited up-to-date research has been done about gueuze production (6–10, 23–26). One later study has given insights into the microbiology of gueuze beers by analyzing differently aged gueuzes, although their production batches were not related to each other (7). Gueuze beer of 6 months old unravels a high diversity of yeasts, such as *Brettanomyces bruxellensis*, *Brettanomyces anomalus*, *Pichia membranifaciens*, and *S. cerevisiae*, and a bacterial community that consists of *Pediococcus damnosus* solely. These microorganisms reflect the microbiota of the maturation phase of lambic beer production processes (1–3). After a few years, the yeast diversity of gueuze decreases, as 2-year-old gueuze beer only contains *B. bruxellensis* and *Brettanomyces custersianus* and gueuze beer 3 years and older contains only *B. bruxellensis*. The bacterium *P. damnosus* has been retrieved from gueuze beers up to 5 years old. This and other studies have also focused on the biochemical changes that occur, in particular concerning the volatile organic compounds (VOCs) that vary during gueuze maturation in bottles (6–10). Esters are the most abundant group of aroma-active VOCs in gueuze beers, especially ethyl esters (6–10). Whereas the concentrations of ethyl acetate and ethyl lactate increase during maturation of gueuze in bottles, the concentrations of other ethyl esters, in decreasing order of abundance those of ethyl octanoate, ethyl decanoate, and ethyl hexanoate, as well as isoamyl acetate concentrations, decrease over time (5, 6). Volatile phenolic compounds, mainly 4-ethylphenol and 4-ethylguaiacol, are always present and contribute to the typical brett flavor of gueuze beers (6, 8–10, 27). Also, organic acids contribute to the perceived flavor of gueuze beers. Non-volatile lactic acid, of which the concentrations typically increase throughout maturation of gueuze in bottles, contributes to a more mild sourness, whereas volatile acetic acid contributes to a sharp acidity (7). However, the concentration of the latter does not change significantly throughout gueuze maturation (7).

Finally, according to the Belgian law, lambic beer is made from water, malted barley, unmalted wheat (at least 30%, m/m), and over-aged hops. Whereas today’s lambic brewers use modern varieties of barley and wheat that are available on the global market, in the past, they depended on the supply from local farmers and markets (28). Nowadays, there is again an increasing interest in old landraces and varieties that were used for lambic beer production in the past. An example is *Zeeuwse Witte*, one of the most cultivated wheat landraces in the 19th century, especially in the southwest province of Zeeland in The Netherlands (29, 30). This wheat is composed of white grains and a white chaff around the kernel. *Zeeuwse Witte* is closely related to the *Witte van Vlaanderen* landrace, which was cultivated a lot in Flanders (Belgium) and belongs to the variety of *Triticum aestivum* var. *albidum*. It was mainly known for its high yield and good (baking) quality. Therefore, the *Zeeuwse Witte* landrace was important for its use in further crop development and, consequently, is at the origin of many modern wheat varieties (31).

The aim of the present study was to map the refermentation and maturation process of blends of lambic beer in bottles for gueuze production for up to 2 years, both microbiologically and biochemically, in particular, to assess its impact on the flavor composition of the resulting gueuze beers. To assess the microbiology throughout this process, both a culture-dependent identification, based on matrix-assisted laser desorption/ionization time-of-flight mass spectrometry (MALDI-TOF MS), and a culture-independent, amplicon-based high-throughput sequencing method were used. The focus of the biochemistry part was on compounds that are typical for the flavor of gueuze beers.

## RESULTS

The production of gueuze beer, composed of a blend of three lambic beers, referred to as L1, L2 and L3, was followed during a period of 2 years. Whereas lambic beer L1 was made with the old landrace *Zeeuwse Witte*, lambic beers L2 and L3 were commonly produced.

### Course of the physicochemical parameters pH and density during gueuze production

**Fig 1:**
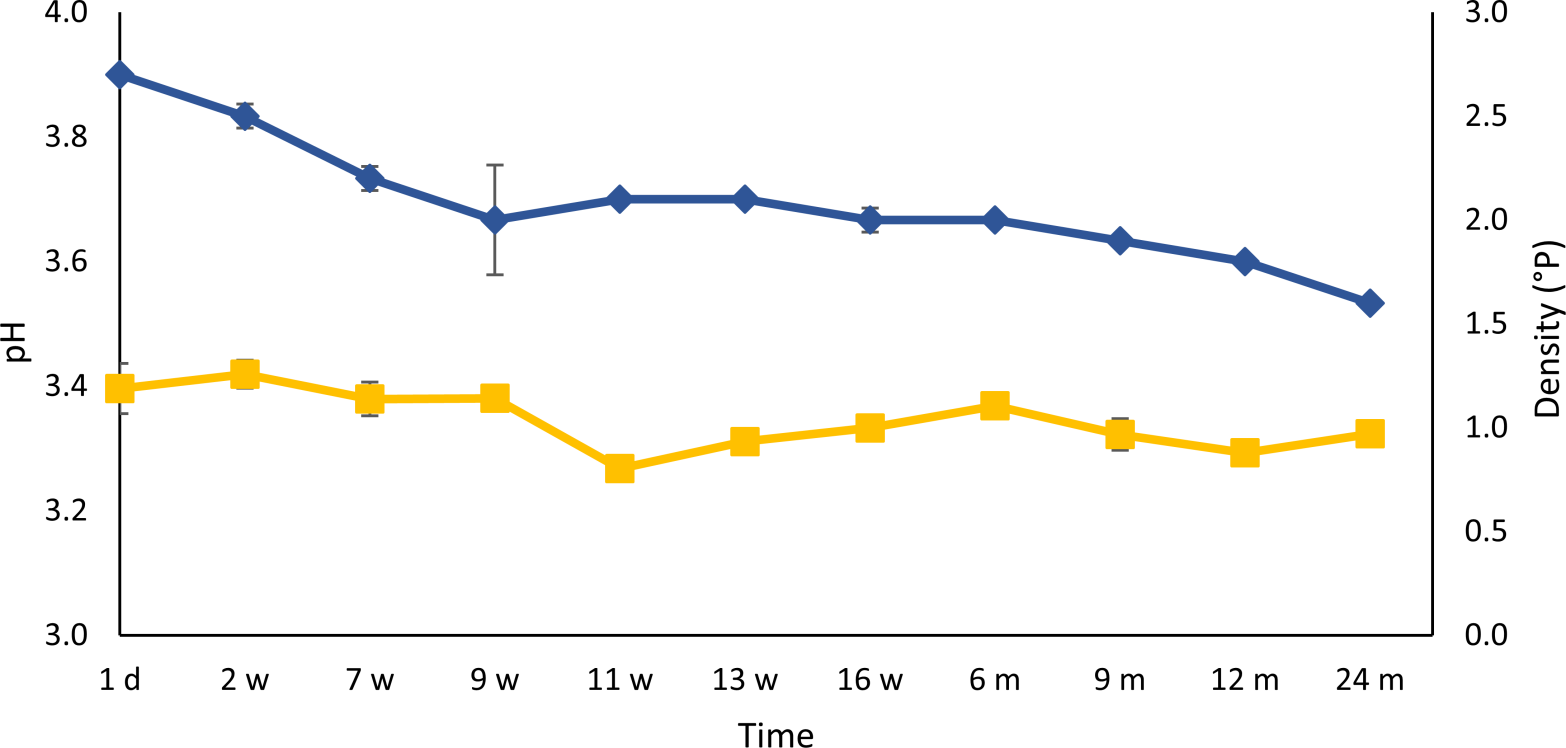
The course of pH (yellow, ■) and density (blue, ◆) during a 24-month gueuze production process in bottles.

Whereas the pH of the gueuze beer did not change significantly throughout the refermentation and maturation process, the density of the beer decreased significantly (*P <* 0.05) from an initial value of 2.7°P to a value of 1.6°P after 24 months (Fig. 1). The steepest density decrease took place during the first weeks of the gueuze production because of the fast consumption of simple saccharides (see below). Later, a slow but steady decrease in density occurred because of the slower consumption of maltooligosaccharides.

### Culture-dependent microbial composition and identities of the lambic beers used for the blend for gueuze production

**Fig 2:**
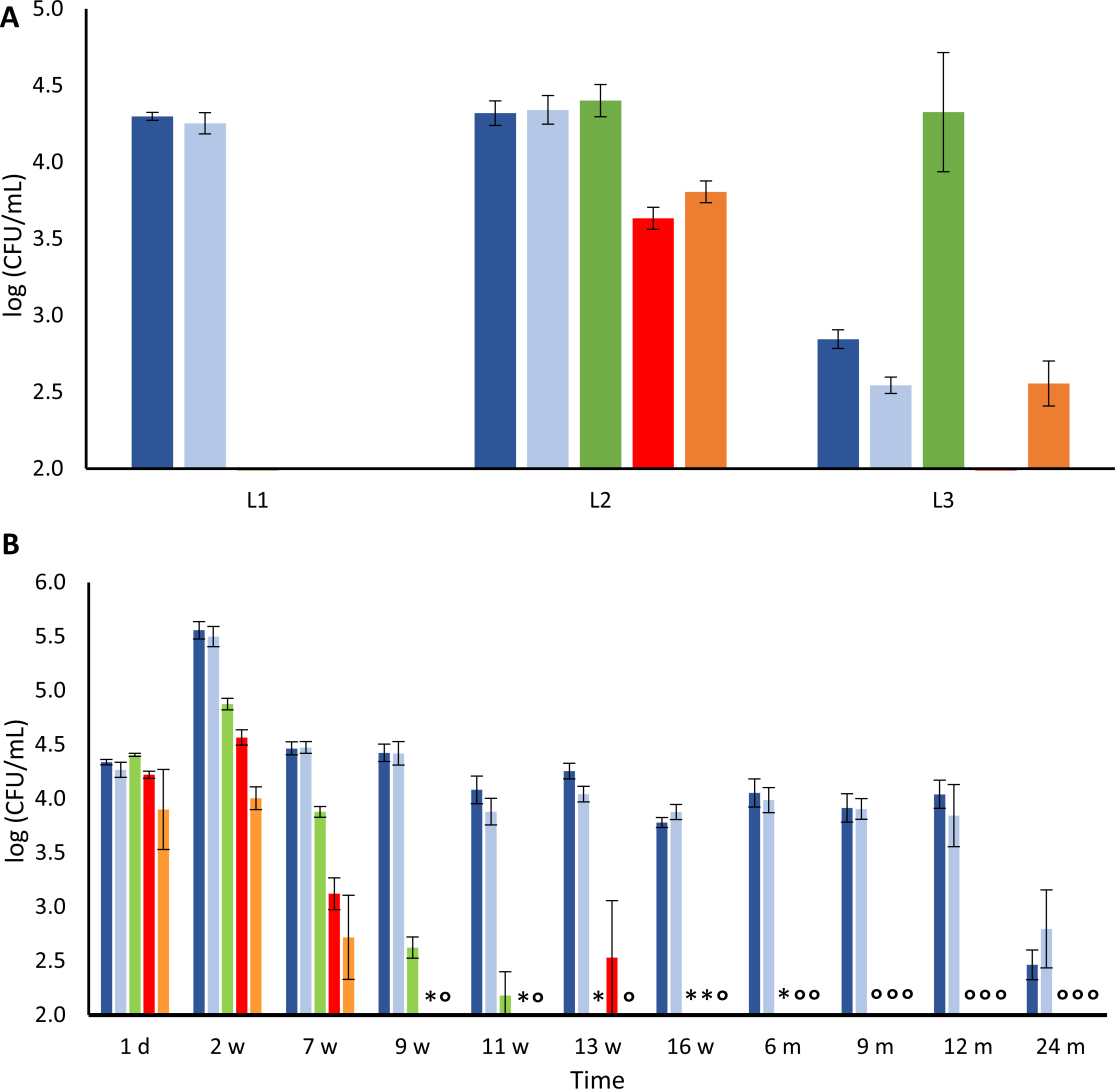
Microbial enumerations of the lambic beers used for the blend for gueuze production, namely, 1-year-old lambic beer L1, 4-year-old lambic beer L2, and 1-year-old lambic beer L3 (**A**) and of the maturing lambic beer blend during a 24-month gueuze production process in bottles (**B**). Presumptive yeasts were enumerated on YPD agar media (dark-blue), presumptive cycloheximide-resistant yeasts on YPDc agar medium (light-blue), presumptive lactic acid bacteria on a modified version of de Man-Rogosa-Sharpe (mMRS) agar medium (red), and presumptive acetic acid bacteria on AAM (red) and a mDMS agar medium (orange). *, under limit of quantification; ○, under limit of detection.

Two 1-year-old lambic beers (L1 and L3) and one 4-year-old lambic beer (L2) were used to make the blend for gueuze production (Fig. 2). In all three lambic beers, the presumptive yeasts were present above the quantification limit, which was not the case for the presumptive bacteria. In lambic beer L1, the presumptive yeasts were the only microbial group above the limit of quantification [4.3 log (CFU/mL) on both yeast-peptone-dextrose (YPD) and YPD containing cycloheximide (YPDc) agar media]. The lambic beer L3 displayed high counts of presumptive lactic acid bacteria (LAB) [4.3 log (CFU/mL)], whereas the counts of the presumptive yeasts and acetic acid bacteria (AAB) were more than 1 log (CFU/mL) lower. Finally, the lambic beer L2 displayed high counts on all agar media used. Especially, the counts on the agar media targeting presumptive AAB, namely, 3.6 log (CFU/mL) and 3.8 log (CFU/mL) on acetic acid agar medium (AAM) and modified deoxycholate-mannitol-sorbitol (mDMS) agar media, respectively, were significantly (*P <* 0.05) higher compared with the other lambic beers.

**Fig 3:**
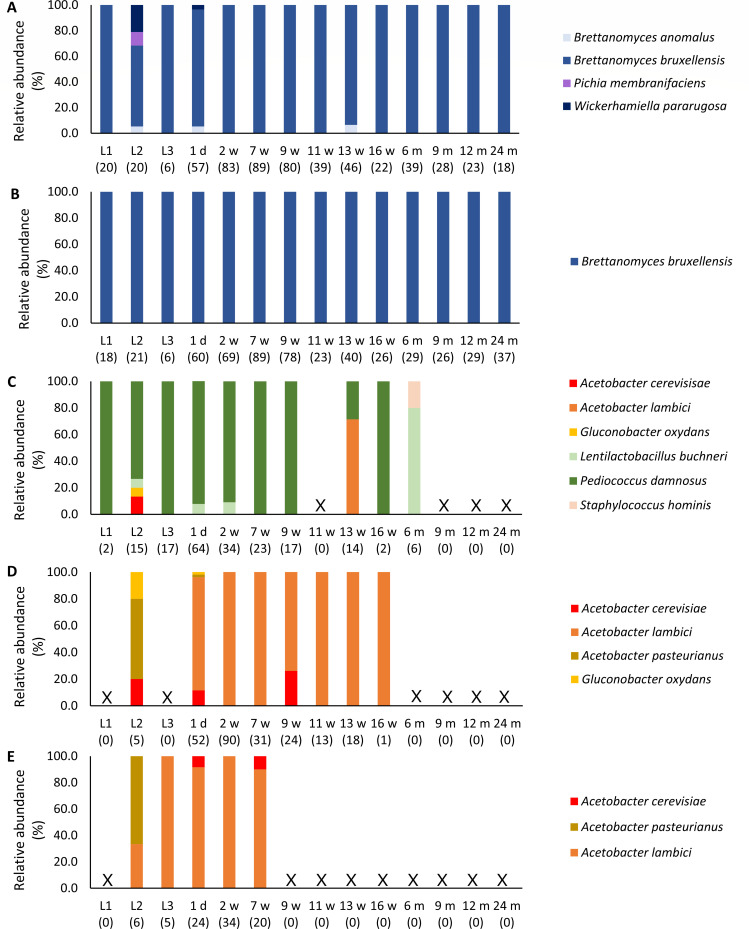
Culture-dependent identification of the microbial isolates randomly picked from agar media on which samples of the three lambic beers used for the blend for gueuze production, namely, 1-year-old lambic beer L1, 4-year-old lambic beer L2, and 1-year-old lambic beer L3, were plated and of the maturing lambic beer blend during a 24-month gueuze production process in bottles. The identifications are given per selective agar medium, namely, YPD agar medium (**A**), YPDc agar medium (**B**), mMRS agar medium (**C**), AAM (**D**), and mDMS agar medium (**E**). The following microbial species were identified: *Acetobacter cerevisiae* (accession no. KF537424.1), *Acetobacter lambici* (accession no. HG329531.1), *Acetobacter pasteurianus* (accession no. KF537405.1), *Brettanomyces anomalus* (accession no. NR_138183.1), *Brettanomyces bruxellensis* (accession no. NR_165974.1), *Gluconobacter oxydans* (accession no. FN391653.1), *Lentilactobacillus buchneri* (accession no. NR_041293), *Pediococcus damnosus* (accession no. NR_042087.1), *Pichia membranifaciens* (accession no. KY104614.1), *Staphylococcus hominis* (accession no. NR_036956.1), and *Wickerhamiella pararugosa* (accession no. AF335965.1). X, no colonies were picked due to lack of growth. The numbers between the brackets show the number of isolates picked from the agar media at the time point under consideration.

The microbial composition, as assessed in a culture-dependent way, showed that lambic beer L2 had not only the highest counts but also the highest diversity of both fungi and bacteria (Fig. 3). Whereas lambic beers L1 and L3 were both characterized by the presence of *B. bruxellensis*, *P. damnosus*, and *Acetobacter lambici*, isolated from YPD and YPDc, mMRS, and AAM and mDMS agar media, respectively, among which the latter species was only present in lambic beer L3, other yeasts and bacteria were also retrieved from YPD, mMRS, AAM, and mDMS agar media for lambic beer L2. Concerning yeasts, in addition to *B. bruxellensis* that was still the most prevalent yeast species in lambic beer L2, *B. anomalus*, *Wickerhamiella pararugosa*, and *P. membranifaciens* were isolated from YPD agar media. For the bacteria, similar findings were noticed. In the case of lambic beer L2, concerning LAB, *Lentilactobacillus buchneri* was isolated from mMRS agar medium in addition to *P. damnosus*, whereas for the AAB, *Acetobacter cerevisiae*, *Acetobacter pasteurianus*, and *Gluconobacter oxydans* were isolated from the AAM and mDMS agar media together with *A. lambici*.

### Course of the microbial enumerations and culture-dependent identities during gueuze production

After blending of the three lambic beers L1, L2, and L3, the counts of the targeted microbial groups were all around 4.0 log (CFU/mL), being 4.3 log (CFU/mL) on both YPD and YPDc agar media, 4.4 log (CFU/mL) on mMRS agar medium, and 4.2 log (CFU/mL) and 3.9 log (CFU/mL) on AAM and mDMS agar media, respectively (Fig. 2). After 2 weeks of gueuze production, the counts on all agar media increased, with the highest increase found on the agar media targeting presumptive yeasts, reaching 5.6 log (CFU/mL) on YPD agar medium and 5.5 log (CFU/mL) on YPDc agar medium. The counts of the agar media used for the bacteria increased to 4.9 log (CFU/mL), 4.6 log (CFU/mL), and 4.0 log (CFU/mL) on the mMRS, AAM, and mDMS agar media, respectively. Thereafter, all counts decreased again. The counts on agar media targeting presumptive AAB dropped below the quantification limit after 9 weeks. The same was found for mMRS agar media, on which the counts dropped below the quantification limit after 13 weeks of gueuze production. The counts on both YPD and YPDc agar media, however, decreased to a value of around 4.0 log (CFU/mL); afterward, they hovered around this same value throughout the first year of the gueuze production. Only after 2 years, the counts on the YPD and YPDc agar media decreased further to final values of 2.5 log (CFU/mL) and 2.8 log (CFU/mL), respectively.

The refermentation and maturation process of the gueuze beers in bottles showed much less microbial diversity, compared with the lambic beers used for the blend. *Brettanomyces bruxellensis*, *P. damnosus*, and *A. lambici* were the most prevalent yeast, LAB, and AAB species identified (isolated from YPD and YPDc, mMRS, and AAM and mDMS agar media, respectively). Only after 6 months of gueuze production, *Lenl. buchneri* became the most prevalent lactic acid bacterium, whereas *P. damnosus* could not be retrieved anymore.

### Culture-independent microbial identifications of the lambic beers used for the blend for gueuze production and during gueuze production

**Fig 4:**
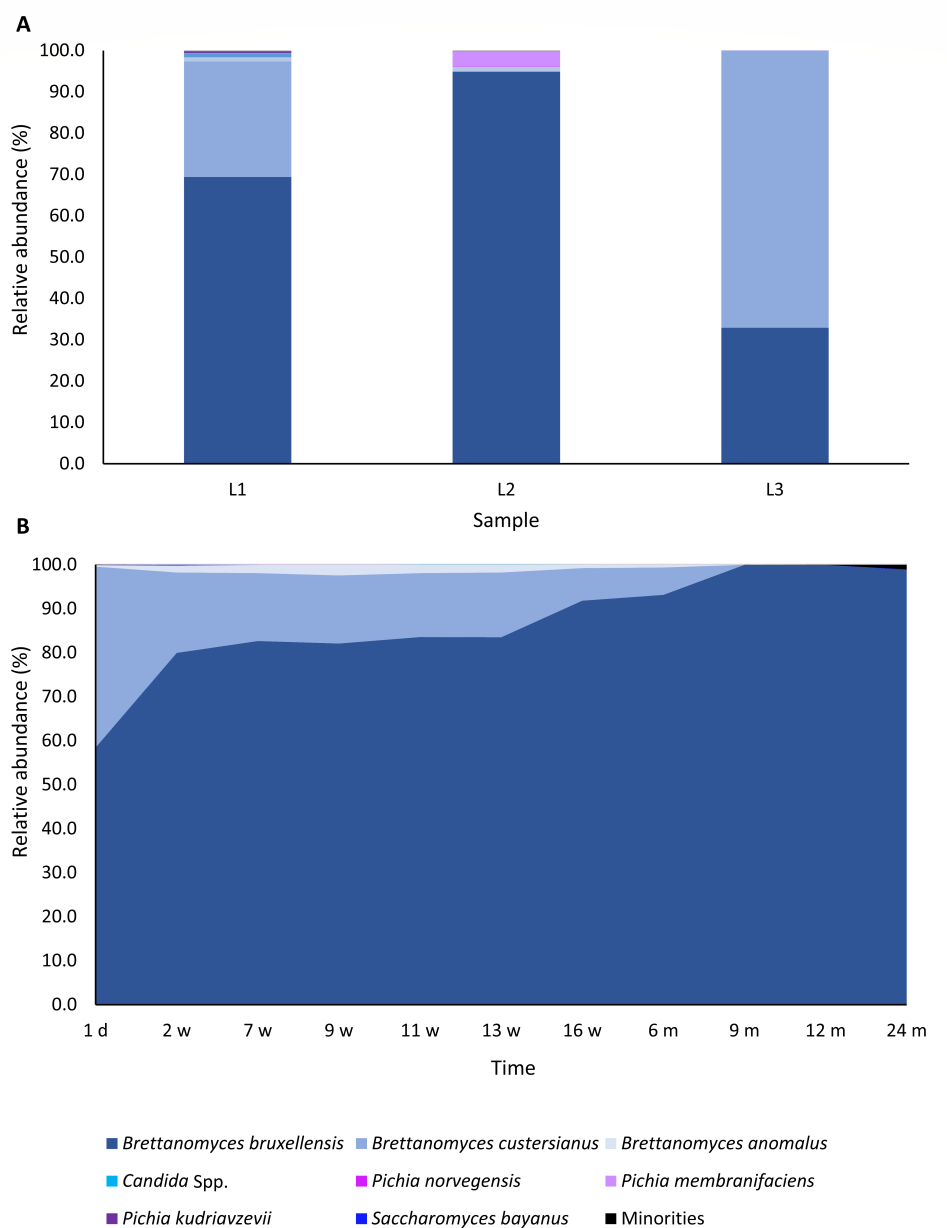
Fungal diversity of the three lambic beers used for the blend for gueuze production, namely 1-year-old lambic beer L1, 4-year-old lambic beer L2, and 1-year-old lambic beer L3 (**A**) and fungal community dynamics encountered during a 24-month gueuze production process in bottles (**B**). The figures show the relative abundance of each yeast species, based on ASVs that were obtained after high-throughput sequencing of the internal transcribed spacer (ITS1) region. The ASVs of the three gueuze samples were taken together to determine these relative abundances at each maturation time point. It is important to notice that the ITS1 regions of the *Brettanomyces* and *Pichia* yeast species were short; therefore, these results should be interpreted with the necessary caution. Reads with a relative abundance lower than 0.05% were categorized as “minorities.”

Based on the sequence reads, the fungal diversity was low, with *Brettanomyces* as the most abundant genus in all lambic beer and gueuze production samples (Fig. 4A; Table 1). The amplicon sequence variants (ASVs) attributed to three different species of this genus, namely, *B. bruxellensis*, *B. custersianus*, and *B. anomalus*, were together responsible for the largest share in the relative abundance of the yeast communities, with a minimum cumulative share of 96.1% in lambic beer L2. Within these species, *B. bruxellensis* was the most abundant one in most of the samples, except for lambic beer L3, for which 67.1% of the reads was ascribed to *B. custersianus*. In lambic beer L2, also a low number of reads (3.7%) was assigned to *P. membranifaciens*. Over time, the relative abundance of *B. bruxellensis* increased, thereby decreasing the diversity in the maturing gueuze, with a relative abundance of 98.9% after 24 months of maturation (Fig. 4B; Table 1).

**TABLE 1:** The alpha diversity of the lambic beer (1-year-old lambic beer L1, 4-year-old lambic beer L2, and 1-year-old lambic beer L3) and gueuze maturation samples, expressed as Simpson (diversity), Pielou (evenness), and Chao1 (true richness) indices

Beer sample	Bacteria			Fungi		
	Simpson	Pielou	Chao1	Simpson	Pielou	Chao1
L1	0.16	0.13	27.38	0.44	0.32	11.00
L2	0.23	0.20	24.47	0.10	0.12	10.00
L3	0.61	0.34	117.00	0.44	0.46	4.00
1 d	0.45 ± 0.02	0.26 ± 0.03	11.07 ± 2.52	0.49 ± 0.01	0.31 ± 0.01	11.00 ± 1.00
2 w	0.46 ± 0.05	0.22 ± 0.04	19.20 ± 12.21	0.33 ± 0.04	0.25 ± 0.03	7.00 ± 1.73
7 w	0.12 ± 0.04	0.10 ± 0.05	11.40 ± 2.94	0.29 ± 0.01	0.24 ± 0.04	6.33 ± 1.15
9 w	0.13 ± 0.02	0.13 ± 0.02	13.46 ± 12.07	0.30 ± 0.08	0.25 ± 0.07	8.33 ± 1.53
11 w	0.07 ± 0.03	0.10 ± 0.03	6.00 ± 0.00	0.28 ± 0.04	0.26 ± 0.02	6.00 ± 1.73
13 w	0.12 ± 0.03	0.12 ± 0.03	15.46 ± 10.62	0.28 ± 0.05	0.26 ± 0.01	6.00 ± 1.00
16 w	0.63 ± 0.00	0.41 ± 0.00	16.00 ± 2.83	0.15 ± 0.02	0.16 ± 0.00	5.67 ± 1.15
6 m	0.57 ± 0.00	0.34 ± 0.00	34.71 ± 6.01	0.13 ± 0.02	0.15 ± 0.03	4.67 ± 1.15
9 m	0.01 ± 0.00	0.01 ± 0.01	15.03 ± 10.84	0.00 ± 0.00	0.00 ± 0.00	1.33 ± 0.00
12 m	0.01 ± 0.00	0.01 ± 0.00	6.34 ± 1.55	0.00 ± 0.00	0.01 ± 0.01	1.67 ± 0.57
24 m	0.00 ± 0.00	0.01 ± 0.00	4.74 ± 3.03	0.02 ± 0.04	0.05 ± 0.05	3.67 ± 2.08

**Fig 5:**
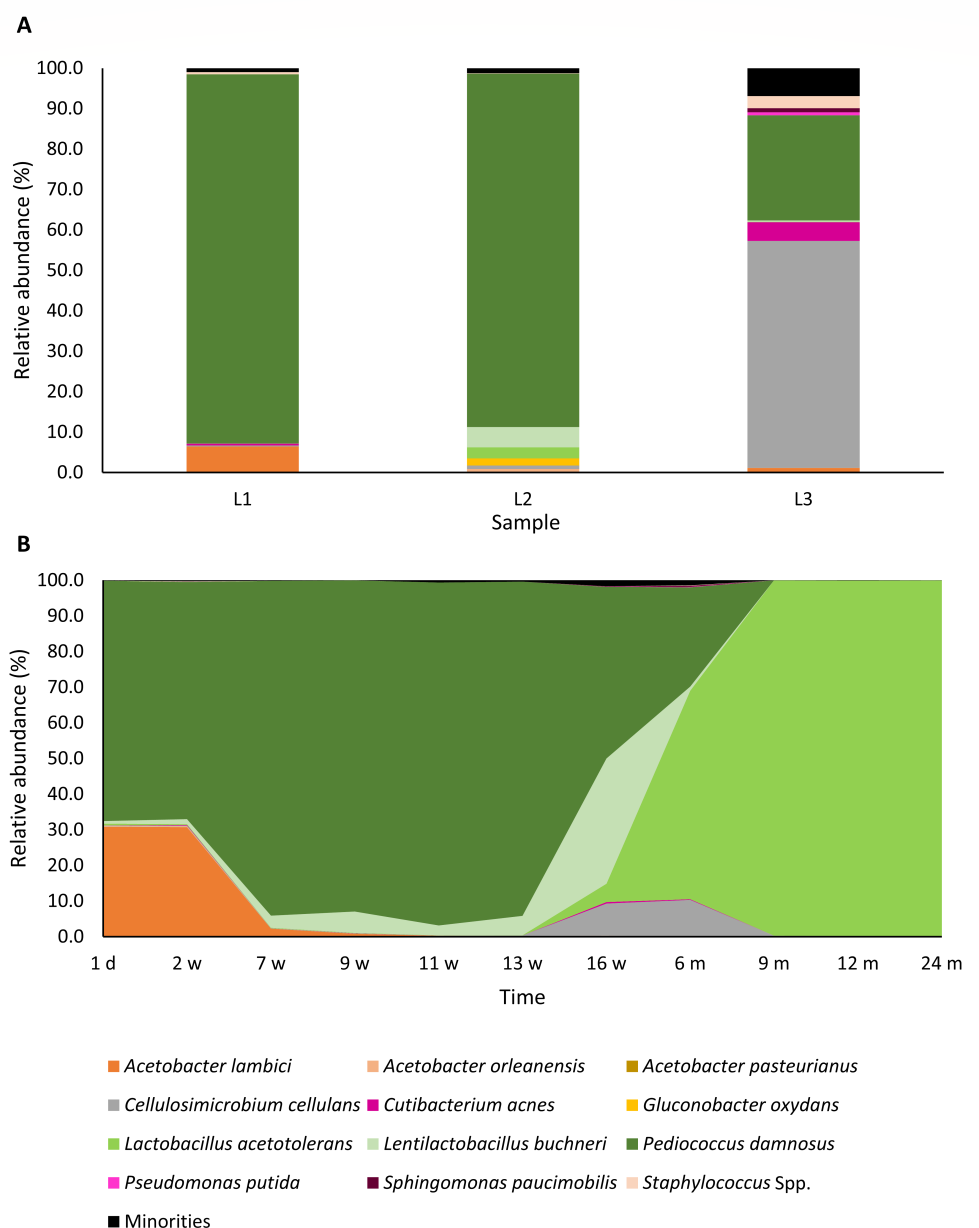
Bacterial diversity of the three lambic beers used for the blend for gueuze production, namely, 1-year-old lambic beer L1, 4-year-old lambic beer L2, and 1-year-old lambic beer L3 (**A**) and bacterial community dynamics encountered during a 24-month gueuze production process in bottles (**B**). The figures show the relative abundance of each bacterial species, based on ASVs that were obtained after high-throughput sequencing of the full-length 16S rRNA gene. The ASVs of the three gueuze samples were taken together to determine these relative abundances at each maturation time point. Reads with a relative abundance lower than 0.5% were categorized as “minorities.”

Concerning the bacterial diversity, *P. damnosus* was, based on the sequence reads, the main species in all three lambic beers used for the blend to make gueuze (Fig. 5A). In lambic beer L2, other LAB were present, namely, in decreasing order of relative abundance, *Lenl. buchneri* (5.0%) and *Lactobacillus acetotolerans* (2.7%). Also, lambic beer L2 showed the highest diversity of the AAB, with reads assigned to *G. oxydans* (1.8%), *Acetobacter orleanensis* (0.7%), and *A. lambici* (0.2%). In contrast, *A. lambici* was the main AAB species in lambic beers L1 and L3. The highest bacterial diversity was found in lambic beer L2, owing to a large share of minorities, which consisted of many different species (Table 1).

*Pediococcus damnosus* remained the most abundant LAB species throughout the first weeks of the gueuze production, increasing in relative abundance until 11 weeks of maturation, during which 96.2% of the reads was assigned to this species (Fig. 5B). Also, the relative abundance of *A. lambici* was high at the start of the gueuze production, with a maximum of 30.9% of the reads assigned to *A. lambici* after 1 day of refermentation, which was followed by a decrease after 2 weeks. From 13 weeks of maturation onward, which was also the time point that the counts on mMRS agar media dropped below the limit of quantification, the relative abundance of *P. damnosus* decreased. Meanwhile, the relative abundance of *Lenl. buchneri* increased, with a maximum of 35.1% of the reads assigned to this species after 16 weeks of gueuze production. Next, from 6 months onward, *Lb. acetotolerans* became the most abundant LAB species, with a relative abundance increasing from 58.5% after 6 months to 99.8% after 24 months of maturation. However, this species was not retrieved in a culture-dependent way.

### Substrate consumption and metabolite production dynamics

#### Carbohydrate concentration dynamics

**Fig 6:**
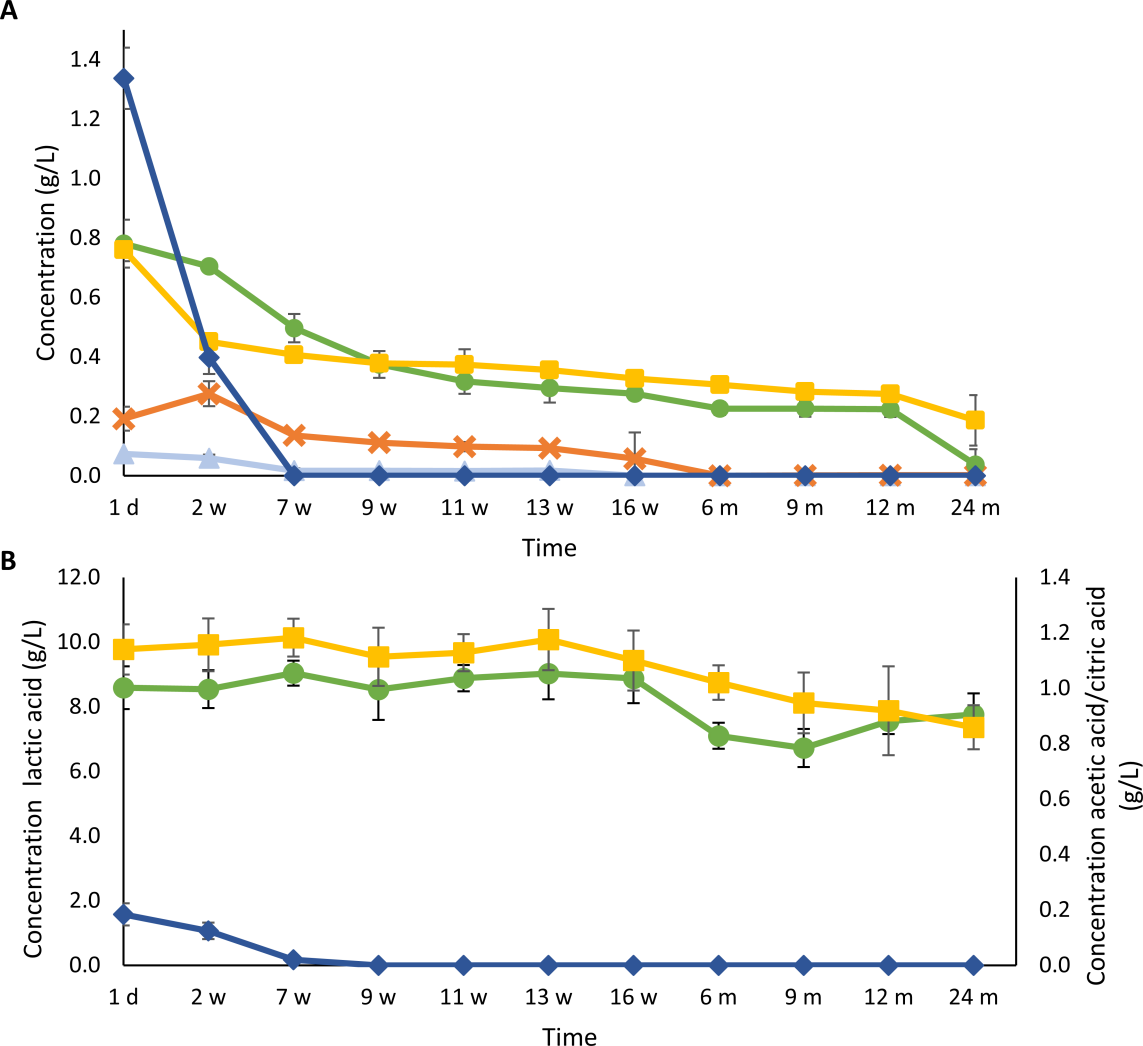
Course of the concentrations of carbohydrates [sucrose (dark-blue, ◆), maltose (light-blue, ▲), maltotriose (orange, ✕), maltotetraose (green, ●), and maltopentaose (yellow, ■)] (**A**) and organic acids [lactic acid (green, ●), acetic acid (yellow, ■), and citric acid (dark-blue, ◆)] (**B**) during a 24-month gueuze production process in bottles.

Sucrose was the main saccharide present in the gueuze beer because of its addition (1.4 g/L; Fig. 6A). After 1 day of refermentation in the bottles, the sucrose concentration was 1.3 g/L, whereas the concentrations of maltose, maltotriose, maltotetraose, and maltopentaose were 73.8 mg/L, 192.0 mg/L, 760.3 mg/L, and 780.7 mg/L, respectively. Glucose and fructose as well as higher maltooligosaccharides, starting from maltohexaose, stayed below the detection limit throughout the whole gueuze production process. After 2 weeks of refermentation, the concentrations of most of the saccharides decreased, except for maltotriose whose concentrations slightly increased, possibly due to a breakdown of higher maltooligosaccharides to maltotriose. The sucrose concentrations decreased significantly during the first 2 weeks of refermentation, from 1.3 g/L to 0.4 g/L, after which it was the first saccharide to be depleted after 7 weeks of maturation. Afterward, also maltose and maltotriose were depleted after 16 weeks and 6 months of maturation, respectively. The concentrations of maltotetraose and maltopentaose decreased at a slower pace. After 24 months of gueuze production, the concentrations of maltotetraose and maltopentaose were 37.3 mg/L and 186.6 mg/L, respectively.

#### Organic acid concentration dynamics

The initial concentration of lactic acid was 8.6 g/L (Fig. 6B). During the first 16 weeks of gueuze maturation, the concentrations hovered around this initial value, after which the concentrations significantly (*P <* 0.05) decreased to 6.7 g/L after 9 months, followed by a significant increase (*P <* 0.05), reaching 7.8 g/L after 2 years. The concentrations of acetic acid stayed around the initial value of 1.1 g/L during the first 13 weeks of gueuze production. After 13 weeks of maturation, this concentration decreased slowly, reaching a final value of 0.9 g/L after 2 years. The initial concentration of citric acid amounted 183.7 mg/L and it was completely consumed after 9 weeks of maturation.

#### Volatile organic compound concentration dynamics

VOC fingerprinting of the beer samples led to 44 organic compounds that were present in two of the three replicates (data not shown), among which ethanol, higher alcohols, esters, phenolic compounds, pyruvate metabolites, and minor compounds (e.g*.* 2,4,5-trimethyl-1,3-dioxolane).

##### Ethanol and higher alcohol concentration dynamics

**Fig 7:**
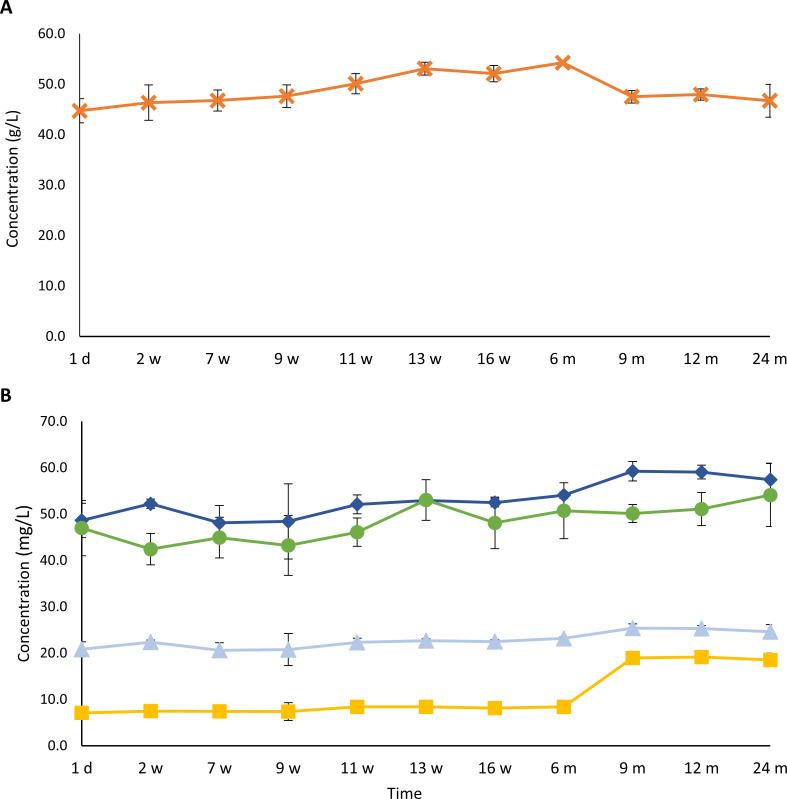
Course of the concentrations of ethanol (**A**) and 3-methyl-1-butanol (dark-blue, ◆), 2-methyl-1-butanol (light-blue, ▲), 2-phenylethanol (green, ●), and 2-methyl-1-propanol (yellow, ■) (**B**) during a 24-month gueuze production process in bottles.

One of the main metabolites that was produced throughout the gueuze production process was ethanol (Fig. 7A). The initial concentration of ethanol was 44.7 g/L. This concentration increased significantly (*P <* 0.05) during the first 6 months of gueuze production, reaching a maximum value of 54.3 g/L. After 6 months of maturation, the concentration decreased again significantly (*P <* 0.05), reaching a final value of 46.7 g/L after 2 years. The concentrations of higher alcohols increased throughout the refermentation and maturation process in the bottles (Fig. 7B). The initial concentrations of 3-methyl-1-butanol, 2-methyl-1-butanol, 2-methyl-1-propanol, and 2-phenylethanol were 48.6, 20.8, 7.1, and 47.0 mg/L, respectively, whereas their concentrations amounted to 57.4, 24.6, 18.5, and 54.1 mg/L, respectively, after 24 months of gueuze production.

##### Ester concentration dynamics

**Fig 8:**
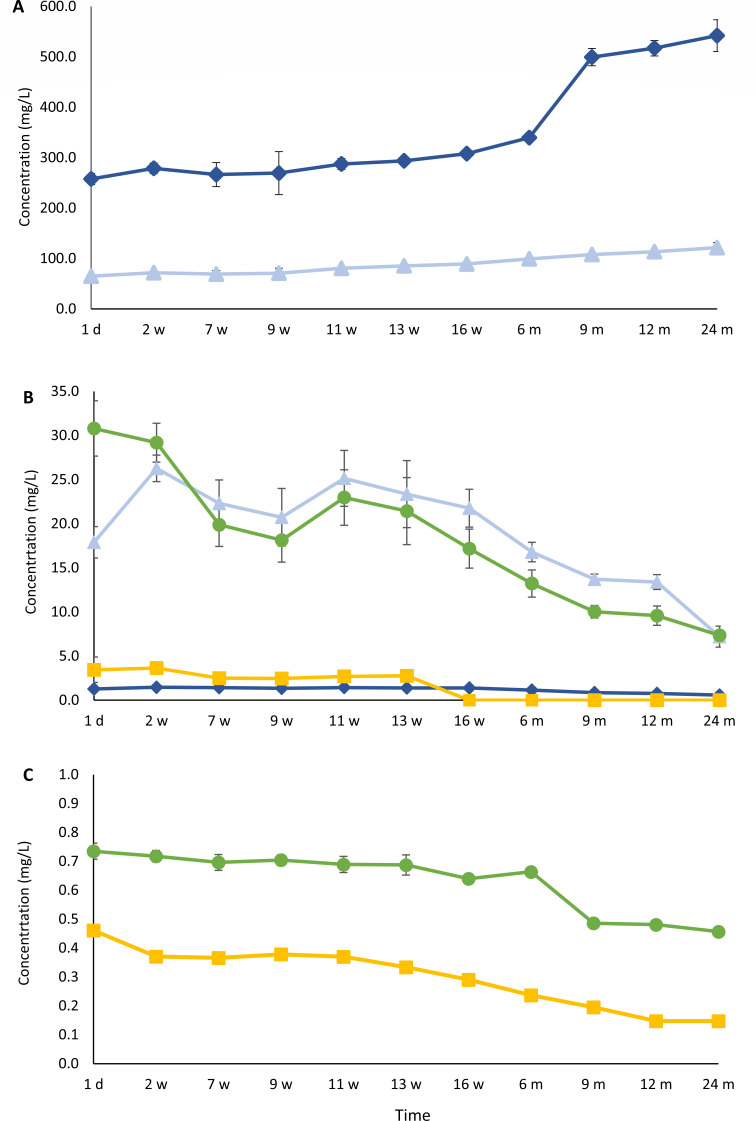
Course of the concentrations of ethyl lactate (dark-blue, ◆) and ethyl acetate (light-blue, ▲) (**A**); ethyl hexanoate (dark-blue, ◆), ethyl octanoate (light-blue, ▲), ethyl decanoate (green, ●), and ethyl dodecanoate (yellow, ■) (**B**); and 2-phenylethyl acetate (green, ●) and isoamyl acetate (yellow, ■) (**C**) during a 24-month gueuze production process in bottles.

The concentrations of the two most abundant ethyl esters, ethyl lactate and ethyl acetate (fruity and floral notes), increased during the gueuze production process (Fig. 8A). The initial concentration of ethyl lactate was 257.8 mg/L, which increased to a final concentration of 542.0 mg/L after 24 months of gueuze production, whereas the concentrations of ethyl acetate increased from an initial concentration of 65.1 mg/L to a final concentration of 121.7 mg/L after 24 months of gueuze production. The initial concentrations of other ethyl esters were—in decreasing order of abundance—30.8 mg/L for ethyl decanoate, 17.9 mg/L for ethyl octanoate, 3.4 mg/L for ethyl dodecanoate, and 1.3 mg/L for ethyl hexanoate, all esters with fruity notes (Fig. 8B). During the first 2 weeks of refermentation in the bottles, the concentrations of most of these esters increased, followed by a decrease that resulted in final concentrations of 7.3 mg/L, 7.2 mg/L, and 0.6 mg/L for ethyl decanoate, ethyl octanoate, and ethyl hexanoate, respectively, after 2 years of gueuze production. The concentrations of ethyl dodecanoate dropped below the limit of detection after 16 weeks of gueuze maturation. Acetate esters showed, in general, a decrease in concentrations during the gueuze production process (Fig. 8C). The concentrations of isoamyl acetate decreased significantly (*P* < 0.05) from 0.4 mg/L to a final value of 0.2 mg/L after 2 years of gueuze production. Also the concentrations of phenylethyl acetate decreased throughout the whole gueuze production process, from an initial value of 0.7 mg/L to a final one of 0.5 mg/L.

##### Volatile phenolic compound concentration dynamics

**Fig 9:**
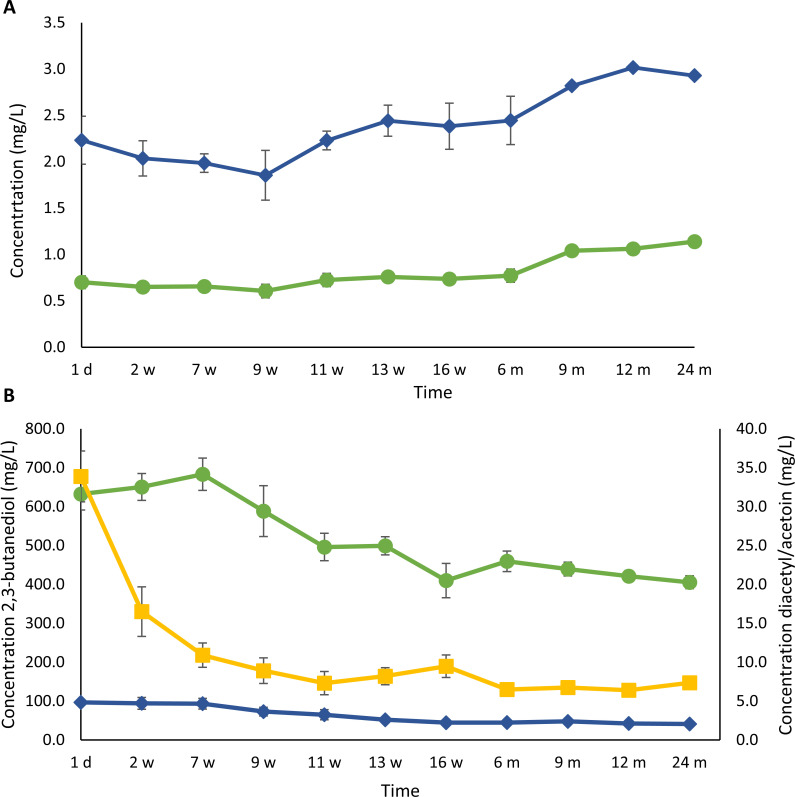
Course of the concentrations of volatile phenolic compounds [4-ethylguaiacol (dark blue, ◆) and 4-ethylphenol (green, ●)] (**A**) and pyruvate metabolites [diacetyl (dark blue, ◆), acetoin (yellow, ■), and 2,3-butanediol (green, ●)] (**B**) during a 24-month gueuze production process in bottles.

The initial concentrations of the volatile phenolic compounds 4-ethylguaiacol and 4-ethylphenol (brett flavor, i.e*.*, smoky, spicy, medicinal, barnyard, horse sweat, and goat-like notes) in the gueuze beers amounted to 2.2 mg/L and 0.7 mg/L, respectively (Fig. 9A). After a slight decrease during the first 9 weeks of refermentation and maturation in the bottles, the concentrations increased again significantly (*P <* 0.05), reaching maximal values of 3.0 mg/L for 4-ethylguaiacol and 1.1 mg/L for 4-ethylphenol after 1 and 2 years of gueuze production, respectively.

##### Pyruvate metabolism compound concentration dynamics

During the gueuze production process in the bottles, the concentrations of compounds related to the pyruvate metabolism, which are responsible for more buttery and creamy flavors and—depending on the concentration—desirable or considered as off-flavor compounds, decreased significantly (*P <* 0.05) (Fig. 9B). The concentrations of diacetyl decreased from 4.8 mg/L to a final value of 2.1 mg/L after 2 years of gueuze production. The concentrations of acetoin decreased more steeply during the first weeks of refermentation in the bottles, from an initial concentration of 33.9 mg/L to a value of 7.3 mg/L after 11 weeks of gueuze maturation, after which the concentration stayed roughly constant around this value until the end of the gueuze production process. Finally, the concentrations of 2,3-butanediol increased during the first weeks of refermentation in the bottles, due to the conversion of diacetyl and acetoin into 2,3-butanediol, reaching a maximal value of 683.5 mg/L after 7 weeks of gueuze maturation, coming from 632.2 mg/L at the start of the gueuze production process. After 7 weeks of maturation, the concentrations of 2,3-butanediol decreased again, reaching a final concentration of 405.7 mg/L after 2 years of gueuze production.

### Biogenic amine concentration dynamics

**Fig 10:**
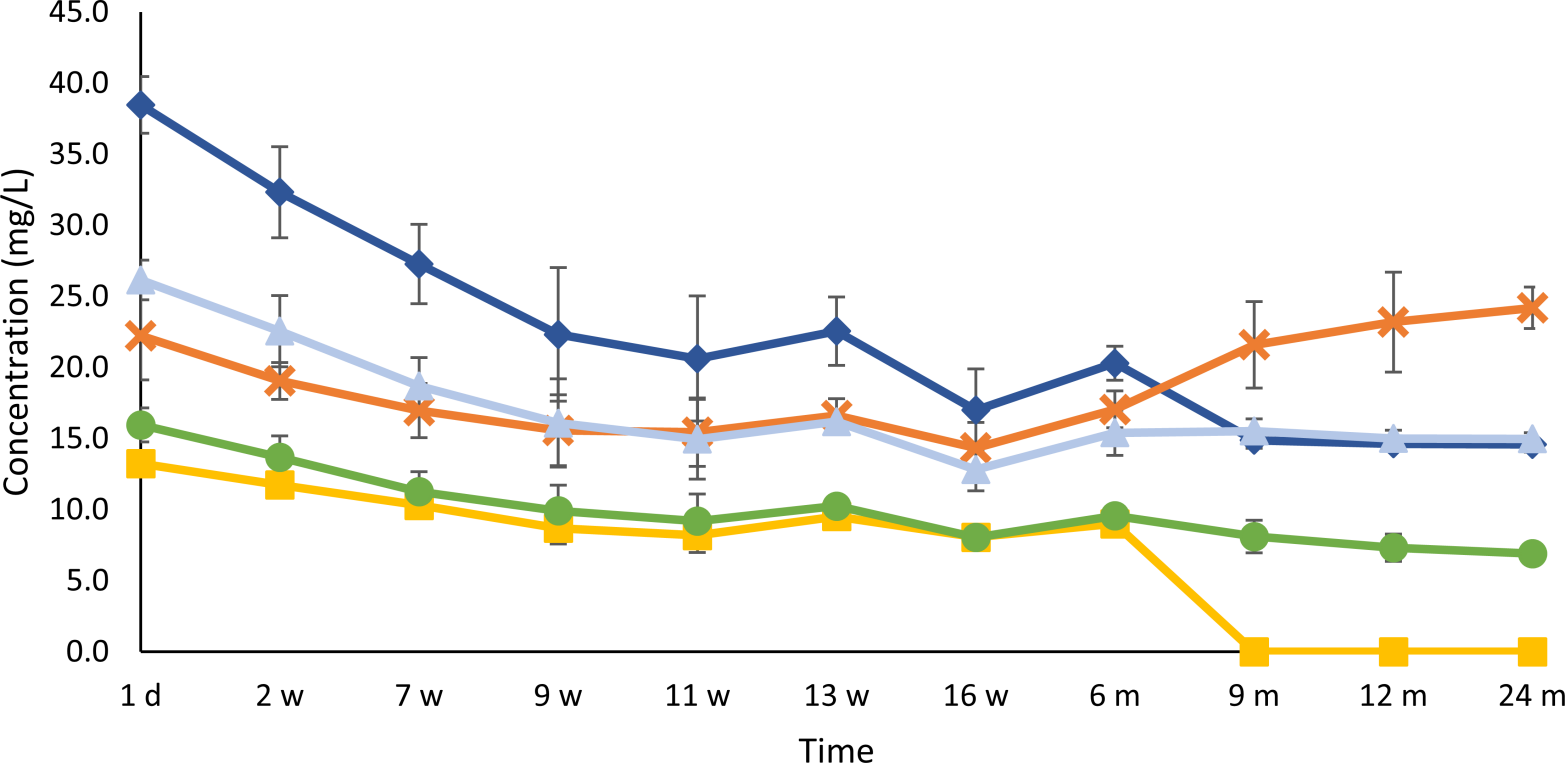
Course of the concentrations of cadaverine (dark-blue, ◆), putrescine (light-blue, ▲), tyramine (orange, ✕), histamine (green, ●), and agmatine (yellow, ■) during a 24-month gueuze production process in bottles.

The concentrations of most of the biogenic amines, namely, cadaverine, putrescine and histidine, decreased significantly during the gueuze production process in the bottles (Fig. 10). Their respective concentrations decreased from 38.5 mg/L, 26.2 mg/L, and 16.0 mg/L to final values of 14.6 mg/L, 15.0 mg/L, and 6.9 mg/L. The concentration of agmatine even dropped below the limit of detection from 9 months of gueuze maturation onward. Finally, the concentration of tyramine also decreased during the first weeks of refermentation in the bottles, namely, from 22.2 mg/L to 12.8 mg/L. However, starting from 16 weeks of gueuze production, the concentration increased again, reaching a final value of 24.2 mg/L after 2 years.

### Sensory analysis

Of the 16 participants of the sensory analysis, five were able to successfully pick the odd sample during a triangle test to compare gueuze beers of 13 weeks and 6 months of maturation. This resulted in a *P* value of 0.66 and, consequently, accepted the null hypothesis stating that there was no significant difference between both samples. This showed that the difference between 13 weeks and 6 months of gueuze maturation was not perceived to be different for an untrained panel. If considering only correct answers, the 6-month beer sample was perceived as slightly less sour but more spicy and woody.

## DISCUSSION

The production of gueuze beers, which are the result of a refermentation and maturation process in glass bottles of a blend of young and old lambic beers, is a strategy for lambic brewers and blenders to cope with the batch-to-batch variability they encounter during the production of lambic beers (1–4, 7). This batch variability is the result of multiple factors, both environmentally and technologically, that influence the lambic beer production process. The most important of these factors is the spontaneous inoculation of the wort with microorganisms originating from the environmental air and the wooden barrels (1, 32, 33). In addition, changes in temperature, pH, and dissolved oxygen concentration during the fermentation and subsequent maturation of lambic beer in horizontal wooden barrels can influence the microbiology and, consequently, the metabolite composition of these beers (34–38). This explains why the blended lambic beers for the gueuze production of the present study differed in microbial composition, not only when they differed in age (lambic beer L2 versus lambic beers L1 and L3) but also when they were of a similar age (lambic beer L1 was characterized by mainly yeasts, in particular *B. bruxellensis*, and *P. damnosus* and *A. lambici* that are characteristic for the maturation of lambic beer, whereas lambic beer L3 showed higher bacterial counts and diversity). The potential of the use of the *Zeeuwse Witte* wheat landrace being responsible for this lambic beer-specific restricted microbial diversity has to be further unraveled. Lambic beer L2 showed the highest diversity, also harboring other LAB (e.g*., Lenl. buchneri*) and yeast (e.g*., P. membranifaciens*) species. The higher counts of AAB in the 4-year-old lambic beer L2 could be explained by the longer maturation time during which oxygen could ingress into the barrel, made possible by the porosity of the wood and, hence, providing microaerobic conditions that could favor the growth of AAB (33, 37, 39).

The present study showed that *Brettanomyces* species characterized the yeast communities of not only the constituting lambic beers but also the gueuze beers produced. The latter contained *B. bruxellensis*, *B. anomalus*, and *B. custersianus*. However, the yeast diversity decreased over time. These *Brettanomyces* yeasts are well adapted to the lambic beer environment, explaining why they are the most abundant yeasts encountered during later stages of the lambic beer production process (33, 36, 38, 40, 41). In gueuze beers, it has been shown before that young beers harbor *B. bruxellensis*, *B. custersianus*, and *B. anomalus*, whereas old ones harbor only *B. bruxellensis* (7). It has indeed been shown that *B. bruxellensis* has an advantage in more anaerobic environments because of the presence of a phenolic acid decarboxylase that contributes to NAD^+^ recovery under these conditions and that is absent in *B. custersianus* (36, 38, 41). A metagenomic study of lambic beer has shown that the gene encoding this enzyme is present in both *Saccharomyces* and *Pediococcus*, whereas its presence is probably strain dependent in *Brettanomyces* wine yeasts (33, 38, 42, 43). In the beginning of the refermentation process of lambic beer in bottles, oxygen was still available, allowing the three *Brettanomyces* species to be present. Throughout the gueuze production process, the oxygen concentrations in the bottles decreased, which gave a competitive advantage to *B. bruxellensis*, hence explaining the shift from several *Brettanomyces* species to *B. bruxellensis* as the most prevalent species during later stages of the gueuze maturation process in bottles. In contrast, during lambic beer production in wooden barrels, the oxygen concentration slightly increases because of the porosity of the wood, explaining a shift from *B. bruxellensis* to *B. custersianus* during the maturation phase (36, 38).

*Acetobacter lambici* and *P. damnosus* were the main bacterial species present during the first weeks of the gueuze production process. Both of these species are indeed very well adapted to the lambic beer environment and, hence, often retrieved from maturing lambic beer (33–38). During bottling, sucrose was added as an additional substrate and oxygen was introduced in the beer because of the mixing, which resulted in an initial growth of the AAB. Upon further refermentation and maturation in the bottles, the dissolved oxygen concentrations in the beer decreased, which affected the growth of *A. lambici*. This made *P. damnosus* by far the most prevailing bacterial species in the maturing gueuze beer. However, this species could only be sporadically isolated after 16 weeks of maturation and, concomitantly, the number of sequence reads assigned to this species decreased drastically. Instead, *Lenl. buchneri* became the most prevailing LAB species, albeit the counts on mMRS agar medium were low and the relative abundance of minor microbial communities increased due to low DNA concentrations extracted from the samples under study. This indicated that the increase in relative abundance of *Lenl. buchneri* was probably not because of the growth of this LAB species, which was most likely in a viable but not culturable (VBNC) state, but rather because of lysis of the *P. damnosus* cells causing lower DNA concentrations of the latter available for extraction. It has been shown before that, after a certain time, *P. damnosus* can not be retrieved from maturing lambic beer, whereas later in the process, sometimes *Lenl. buchneri* prevails (33, 36). This showed a better adaptation of *Lenl. buchneri* to the lambic beer environment or that *P. damnosus* was not well adapted to the circumstances encountered during later stages of the lambic beer production process and, hence, more susceptible to cell lysis. Another explanation could be bacteriocin production by *Lenl. buchneri*, which is bacteriocidal against multiple LAB species, including *Pediococcus acidilactici* (44, 45). Also multiple pediococcal species produce bacteriocins, which in some cases can show autolytic activity (46, 47). However, the latter seems unlikely here, since the bacteriocin produced by *P. damnosus*, pediocin PD-1, has not been shown to be active against other pediococci and is not very active at low pH values (48–50). Also, phage infections could not be excluded. In the genome of *P. damnosus* LMG 28219 isolated from a mature Belgian red-brown acidic ale, an intact prophage region corresponding with the phage *Sha1* has been predicted (51). This phage was first isolated from kimchi, in which it infected *Lactiplantibacillus plantarum* (52). The decrease of the cell viability of *P. damnosus*, due to a change from good environmental conditions in the lambic beer to more stressful conditions and nutrient depletion after a few weeks of refermentation in the bottles, might have led to prophage induction (53, 54). The growth of *Lb. acetotolerans* after lysis of the *P. damnosus* cells caused outcompetition of *Lenl. buchneri*, which is a slow grower (55). However, this did not lead to an increase of the counts on mMRS agar media, which is in accordance with the fact that *Lb. acetotolerans* is one of the most difficult common beer spoilage LAB species to cultivate (56). It requires Tween 80, which was not present in the mMRS agar medium used in the present study, it does not grow at low temperatures (the mMRS agar medium was incubated at 20°C in the present study), and it is very sensitive to oxygen (strict anaerobic incubation was not applied in the present study), which may explain the occasional isolation of *A. lambici* from mMRS agar medium (56, 57). The aforementioned criteria were also a possible explanation why *Lb. acetotolerans* was not competitive against *P. damnosus* in the lambic beer environment. Indeed, at the start of the refermentation process in the bottles, similar conditions as those encountered during lambic beer production in the barrels prevailed, enabling *Lb. acetotolerans* to remain in a VBNC state. Later, the oxygen concentrations decreased, the *P. damnosus* cells lysed, and hence, new nutrients were released into the beer, which, in combination with high acetic acid concentrations to which *Lb. acetotolerans* is tolerant and a higher temperature during the refermentation process in bottles compared with the lambic beer production process, resulted in the growth of *Lb. acetotolerans* (58).

Refermentation and maturation of lambic beer in bottles for the production of gueuze result in many biochemical changes that can influence multiple properties of the final beer. First, this process increases the microbial and biochemical stability of the beer (18–20). The depletion of saccharides and the formation of mainly ethanol during the first weeks of the refermentation process in the bottles resulted in a decrease of the dissolved oxygen concentrations and an increase of the carbon dioxide concentrations, all contributing to the antimicrobial hurdles present in the beer (19, 20). This in turn had a negative influence on the bacteria present during this period, whereas the yeasts withstood these selective pressures. *Brettanomyces* yeast species are indeed tolerant toward many of these barriers thanks to, for instance, a make-accumulate-consume strategy for both ethanol and acetic acid (33, 36, 38, 40, 41, 59). Whereas the production of ethanol and acetic acid by the *Brettanomyces* yeasts first outcompeted the other microorganisms present in the lambic beer blend, they again consumed these compounds to sustain their survival in this environment. Indeed, once exhaustion of the carbohydrates, especially sucrose, maltose, and maltotriose, occurred and no bacteria were found anymore, which was the case when after 16 weeks of gueuze production, both ethanol and acetic acid were consumed. Onward, the concentrations of other *Brettanomyces* yeast-related compounds, such as higher alcohols, volatile phenolic compounds, and some ethyl esters, further increased, confirming that these yeasts were still active. Although the make-accumulate-consume strategy occurs under aerobic conditions, the present study showed that it also happened during gueuze production in bottles, likely because of microaerobic conditions (no exhaustion of the oxygen in the bottles or further air ingress into the beer because of the permeability of the cork) or taking place at the liquid/air interphase (which is quite large because of the horizontal position of the bottles).

Refermentation and maturation of lambic beer in bottles for gueuze production serve as a fine-tuning process for its quality before consumption, hereby focusing on flavor, besides other organoleptic properties, such as carbonation of the beer (14–18). The characteristic flavor compounds of gueuze are the same as those of lambic beer, whereby their concentrations further increased or decreased during maturation of the beer in the bottles. For instance, the sourness of the gueuze beers did not increase throughout the refermentation and maturation process in the bottles. This was in contrast with increasing concentrations of lactic acid with an increasing age of gueuze beers shown before (7). Yet, both lactic acid (produced consecutively by *P. damnosus* and *Lb. acetotolerans*), responsible for the soft, fresh acidity, and acetic acid (produced by mainly *A. lambici*), responsible for the sharp acidity, were produced in the gueuze beers of the present study, but these organic acids were almost immediately converted into their respective ethyl esters. This might also explain why, from the moment that both the LAB and AAB counts dropped below the detection limit, the concentrations of these organic acids decreased, while the concentrations of the esters ethyl lactate (buttery, fruity) and ethyl acetate (solvent like, fruity) increased. During the first weeks of refermentation of the lambic beer blend, both LAB and AAB encountered favorable conditions, namely, high fermentable concentrations of carbohydrates for the production of both lactic acid and acetic acid and the presence of both oxygen and ethanol for the production of acetic acid, respectively (2). In addition, *Brettanomyces* yeasts produced acetic acid in the presence of oxygen from both glucose and ethanol (41, 59, 60). Whereas the concentrations of the two most abundant ethyl esters, ethyl lactate and ethyl acetate, increased, those of the other esters decreased over time. The concentrations of acetate esters, such as isoamyl acetate (banana) and 2-phenylethyl acetate (honey), are indeed typically low in lambic and gueuze beers (6, 7, 36, 61). This is due to the isoamyl acetate-hydrolyzing esterase *IAH1* activity of *Brettanomyces* yeasts on multiple acetate esters (61–65). Furthermore, these yeasts do not possess acetyltransferase genes, such as *ATF1* and *ATF2*, that are necessary for the production of acetate esters (62–64). Moreover, the concentrations of both isoamyl acetate and 2-phenylethyl acetate stayed below their respective beer flavor thresholds throughout the entire gueuze production process (66). However, the increase of the concentration of one of the acetate esters, ethyl acetate, throughout the gueuze production process of the present study, likely owed to its formation through other multiple ways. Indeed, ethyl acetate can be produced through both ethanol acetyltransferase (EAT) and ethanol O-acyltransferase activities, the latter being encoded by the genes ethanol hexanoyl transferase I (*EHT1*) and ethyl ester biosynthesis (*EEB1*) (67, 68). However, up to now and to the best of the authors’ knowledge, the presence of these genes encoding ethyl ester production has not been shown in *Brettanomyces* yeast species, except for one study describing an EHT1 homolog, although the ester profile of lambic beers is characterized by mainly ethyl esters and is mainly determined by *Brettanomyces* yeasts (6–10, 62). The former hypothesis is supported by the fact that in both a previous study and the current study, the concentrations of other ethyl esters also mainly decreased throughout gueuze maturation, indicating that these esters were possibly not formed by *Brettanomyces* (7). Also, the possibility and the extent to which (ethyl) esters are produced are species and strain dependent (62). Furthermore, a metagenomic study has assigned esterase genes to AAB and, consequently, the possible production of esters by AAB (38). Nevertheless, also the chemical formation and degradation of ethyl esters could not be excluded, which is supported by high and increasing concentrations of ethyl acetate and ethyl lactate in an environment with high concentrations of ethanol, acetic acid, and lactic acid. Among the higher ethyl esters, ethyl octanoate (apple), ethyl decanoate (grapes), and ethyl hexanoate (apple) display, in a decreasing order of abundance, the highest concentrations in lambic beers as well as in wine (6, 7, 69). Moreover, their decreasing concentrations were, after 2 years of gueuze production, still above their flavor thresholds, contributing to, in addition to ethyl acetate and ethyl lactate, the typical fruity taste of gueuze beers (66).

Also, the concentrations of the volatile phenolic compounds, 4-ethylphenol (smoky notes) and 4-ethylguaiacol (spicy and medicinal notes), varied during gueuze production. They were above the flavor threshold throughout the entire refermentation and maturation process followed in the bottles (70). A decrease during the first weeks of refermentation in the bottles was followed by an increase from 9 weeks onward, which had to be ascribed to the Custers effect, as there was a switch from aerobic to anaerobic/microaerobic conditions upon refermentation and maturation in the bottles (41, 71). In the presence of oxygen at the start of the refermentation in the bottles, high concentrations of acetic acid and ethanol were produced by the *Brettanomyces* yeasts, as part of their make-accumulate-consume strategy mentioned above. This was accompanied by an increase in biomass. The increase in biomass and acetic acid concentrations required the cofactor NAD^+^. In the presence of oxygen, NAD^+^ is regenerated through the production of ethanol, which is in contrast to other yeasts that prefer respiration over fermentation under aerobic conditions with high substrate concentrations. This Custers effect assures that the other processes mentioned above could continue. However, once a switch is made toward anaerobic conditions, NAD^+^ can not be regenerated, also since these yeasts are not able to produce glycerol that can help with this regeneration, which will stop the glycolysis and introduce a lag phase (71). Only upon the onset of slower processes to regenerate NAD^+^, the glycolysis can again take place and, in turn, biomass, ethanol, and acetic acid can be produced again. One of these slower routes to regenerate NAD^+^ is the production of volatile phenolic compounds, since the enzyme vinyl phenol reductase that is responsible for the conversion of 4-vinyl compounds into their respective 4-ethyl compounds uses NADH + H^+^ as the cofactor, which then gets oxidized to NAD^+^ (72). Also acetoin can be used as an alternative electron acceptor to regenerate NAD^+^ (36–38, 41).

Finally, the refermentation and maturation process in the bottles contributed to a reduction of the concentrations of compounds that can negatively influence the taste and aroma of (gueuze) beers. Such a first group of compounds originated from the pyruvate metabolism and contributed to more buttery and creamy flavors, namely, diacetyl, acetoin, and 2,3-butanediol. During the first weeks of the gueuze production process, the conversion of mainly acetoin and, to a lesser extent, diacetyl into 2,3-butanediol, which has a flavor threshold that is much higher compared with the former two compounds, took place. Nevertheless, these diacetyl concentrations stayed above their flavor threshold, which is typically around 0.05 mg/L in lager beers (73). However, these high diacetyl concentrations were masked by the higher complexity of the taste of gueuze beers compared with that of lager beers. The decrease of the 2,3-butanediol concentrations after 7 weeks of gueuze maturation could be ascribed to the conversion of 2,3-butanediol into acetoin, which is in turn consumed by the *Brettanomyces* yeast species to regenerate NAD^+^ (36–38, 41, 74, 75). Alternatively, other chemical reactions, such as dehydration, esterification, or ketalization, could have taken place (75). With regard to the latter reaction, 2,4,5-trimethyl-1,3-dioxolane (phenolic notes and astringent) was formed during later stages of the gueuze production process. This is an acetal formed through the reaction of 2,3-butanediol with acetaldehyde and is often found in trace concentrations in beers (typically lower than 1 mg/L), with a slight impact on their flavor because of the concomitant threshold value (18, 76). Finally, also the concentrations of biogenic amines, another group of unwanted compounds that may occur in lambic beer (32, 33, 36), mainly showed a decreasing trend during the gueuze production process. Some yeasts and bacteria produce amine oxidases, which convert biogenic amines into aldehydes, hydrogen peroxide, and ammonium (77). These enzymes occur in multiple LAB, including pediococcal species that have been isolated from a wine-related environment (78, 79). Since amine oxidases require oxygen for the regeneration of the coenzyme FAD, the degradation of biogenic amines could only take place under aerobic conditions (77), which was the case during the first weeks of refermentation in the bottles. The increase of the concentration of tyramine during the last months of the gueuze production process of the present study may be ascribed to its liberation from lysed *P. damnosus* cells, since this species is a known producer of tyramine during lambic beer productions (32, 33, 36, 38). Lysis of the cells releases the intracellular tyrosine decarboxylase into the beer to convert tyrosine into tyramine. However, *Lenl. buchneri* and *Lb. acetotolerans* could not be excluded as tyramine producers, since many LAB are known producers of biogenic amines (80, 81).

To conclude, during the present study, the microbiological and biochemical changes that occurred during a bottle refermentation process of lambic beers to produce gueuze were followed over time. The first half year of the gueuze production process in the glass bottles was characterized by a high and changing microbial diversity, corresponding with an initial increase of the counts of all microbial groups. This mainly reflected the maturation phase of the constituting lambic beers that were produced in wooden barrels. This was followed by a decrease of mainly the bacterial counts, the sequential occurrence of three LAB species, namely, *P. damnosus*, *Lenl. buchneri*, and *Lb. acetotolerans*, and the decreasing diversity of yeasts. The end of the maturation process in the bottles was much less diverse microbiologically, with *B. bruxellensis* and *Lb. acetotolerans* as most abundant yeast and bacterial species, respectively. Mainly, the metabolism of the yeasts was responsible for the biochemical changes that occurred during the gueuze production process. The acidity decreased, partially due to the conversions of acetic acid and lactic acid into their respective ethyl esters and possibly also because of the make-accumulate-consume strategy displayed by the yeasts. Whereas the concentrations of most metabolites related to the metabolism of the *Brettanomyces* yeast species increased over time, such as those of higher alcohols, volatile phenolic compounds, and the ethyl esters ethyl acetate and ethyl lactate, the concentrations of other VOCs decreased, being mainly higher ethyl esters, acetate esters, and pyruvate metabolites, all impacting the final flavor of the gueuze beers. Moreover, most of these changes, both microbiologically and biochemically, could be linked to the switch of a nutrient- and oxygen-rich environment to one with lower concentrations of nutrients and dissolved oxygen. Finally, the characteristics of the gueuze beers followed during the present study met the legal requirements of *oude geuze*.

## MATERIALS AND METHODS

### Experimental set-up

To follow gueuze beer production over time, a blend of three lambic beers, made by three different lambic breweries (A, B, and C) that were all located in the southwest region of Brussels, was made in brewery A. Lambic beer 1 (L1) was produced by brewery A and was 1 year old at the moment of blending. This lambic beer was produced with the old landrace *Zeeuwse Witte* and had a pH of 3.5 [measured with an InoLab 720 pH-meter (WTW, Weilheim, Germany)] and a density of 2.5°P [measured with a DMA 35 (Anton Paar, Graz, Austria)]. The second lambic beer (lambic beer 2, L2) that was added to the blend was commonly produced by brewery B, was 4 years old, and had a pH of 3.4 and a density of 1.9°P. Lambic beer 3 (L3) was 1 year old, had a pH of 3.5 and a density of 3.3°P, and was commonly produced by brewery C. Finally, 4.0 kg of sucrose was added to the blend, which consisted of 35% (vol/vol) L1, 40% (vol/vol) L2, and 25% (vol/vol) L3, resulting in a mixture with a total volume of 2,600 L and an average density of 2.7°P. An aliquot part of this mixture was then distributed between 33 bottles of 750 mL, allowing analyses at 11 different time points, which were then closed with a cork, and incubated horizontally at a temperature of 20°C.

### Sampling

After 1 day; 2, 7, 9, 11, 13, and 16 weeks; and 6, 9, 12, and 24 months, three bottles, which were considered as biological triplicates, were withdrawn for sampling. Therefore, the bottles were carefully inverted a few times to homogenize the beer, after which three samples of 50 mL were taken per bottle. A first sample was used for a culture-dependent analysis, both colony enumeration and identification. Another sample of 50 mL was centrifuged at 4,696 × *g* and 4°C for 20 min. The cell-free supernatant was separated from the cell pellet, and both were stored at −20°C until further culture-independent and metabolite target analysis, respectively. In addition, samples (three times 50 mL) of the three lambic beers that were used to prepare the blend (L1, L2, and L3) were taken and analyzed in the same way as the beer samples mentioned above.

### Physicochemical parameters

Of each beer sample, the pH was measured with an InoLab 720 pH-meter (WTW) and the density (expressed in °P) was measured with a DMA 35 (Anton Paar).

### Culture-dependent enumeration and identification

#### Selective plating and incubation

Each beer sample was subjected to a culture-dependent microbiological analysis through selective plating and incubation to determine the microbial community dynamics, as described previously (34–37). Therefore, each sample was 10-fold diluted in saline [8.5 g of sodium chloride (Merck, Darmstadt, Germany) in 1 L of ultrapure water (MilliQ; EMD Millipore, Billerica, Massachusetts USA)], followed by plating of 100 µL on six different agar media. Yeast extract-peptone-dextrose (YPD) agar medium [5 g/L of yeast extract (Oxoid, Basingstoke, Hampshire, UK), 10 g/L of bacteriological peptone (Oxoid), and 20 g/L of dextrose (Merck)] was used to target presumptive yeasts (34–36). YPDc agar medium, which is YPD agar medium supplemented with 50 ppm of cycloheximide (Sigma-Aldrich, Saint-Louis, Missouri, USA), was used to target presumptive cycloheximide-resistant yeasts (34–36). Both agar media were supplemented with 100 ppm of chloramphenicol (Sigma-Aldrich) to prevent the growth of bacteria. A mMRS agar medium [52 g/L of MRS (Oxoid) supplemented with 1 mL/L of a solution consisting of 200 ppm of each of the vitamins cobalamin, folic acid, nicotinic acid, pantothenic acid, pyridoxal phosphate, and thiamine (all from Sigma-Aldrich)] (34–36) was used to target presumptive LAB. Two agar media were used to target AAB. The first agar medium was a mDMS agar medium [10 g/L of bacteriological peptone (Oxoid), 3 g/L of yeast extract (Oxoid), 1 g/L of potassium dihydrogen phosphate (Merck), 1 g/L of dextrose (Merck), 1 g/L of mannitol (Merck), 1 g/L of sorbitol (VWR International, Radnor, Pennsylvania, USA), 0.1 g/L of sodium deoxycholate (Sigma-Aldrich), 0.03 g/L of bromocresol purple (Merck), 0.02 g/L of magnesium sulphate heptahydrate (Merck), 6.3 g/L of lactic acid (Sigma-Aldrich), and 5 g/L of absolute ethanol (Fisher Scientific, Loughborough, Leicestershire, UK; added after autoclaving), pH of 4.7] (37, 82). The second agar medium was AAM [10 g/L of dextrose (Merck), 15 g/L of bacteriological peptone (Oxoid), 8 g/L of yeast extract (Oxoid), 3.15 g/L of acetic acid (Merck), 3.9 g/L of absolute ethanol (Fischer Scientific), and 3.4 g/L of hydrogen chloride (1 M; Merck); the latter three compounds were aseptically added after autoclaving of the medium] (34, 35). To the agar media targeting LAB and AAB, 200 ppm of cycloheximide and 5 ppm of amphotericin B were added to prevent the growth of fungi. Beers from bottles withdrawn after 1 day of refermentation were also plated on RAPID’*Enterobacteriaceae* agar medium (RAPID Entero; Bio-Rad, Marnes-La-Laquette, France) and incubated at 37°C for 24 h to check if presumptive enterobacteria could be cultivated. All agar media were prepared with 15 g/L of agar (technical agar; Oxoid), except for mDMS, to which 20 g/L of agar was added. The YPD, YPDc, mDMS, and AAM agar media were incubated aerobically at 28°C for 7 days. The mMRS agar media were incubated at 20°C for 10 days, which was performed anaerobically [BD BBL Gaspak 150 anaerobic incubator (BD Biosciences, San Jose, California, USA) harboring AnaeroGen 2.5L catalysts (Thermo Scientific, Waltham, Massachusetts, USA)] to prevent the growth of AAB (36).

#### Clustering and identification of the isolates with MALDI-TOF-MS

After colony enumeration, 10% of the colonies, with a minimum of six colonies, was picked from agar media corresponding with a dilution harboring between 30 and 300 colonies and subcultivated twice on their respective agar medium (36, 37). Afterward, the third-generation colonies were used for dereplication and identification with MALDI-TOF-MS. First, cultures of these colonies were prepared for preservation at −80°C. For yeasts retrieved from both YPD and YPDc agar media, a liquid version of YPD medium (without agar) was used. LAB were grown in liquid MRS (Oxoid) medium. AAB retrieved from both mDMS and AAM agar media were grown in a liquid medium that resembled that of AAM but without hydrogen chloride, acetic acid, and ethanol. After overnight incubation at 30°C, aliquots of 1.6 mL of the cultures were transferred to cryovials, which contained 0.4 mL of glycerol (Sigma-Aldrich). Second, to prepare samples for MALDI-TOF-MS analysis, an inoculation loop of the third-generation colonies was resuspended in 300 µL of MilliQ (36, 37, 83), to which 900 µL of absolute ethanol (Fisher Scientific) was added. After mixing by sample inversion, the samples were microcentrifuged (21,100 × *g*, 3 min, 4°C) and stored at −24°C. For the actual analysis, a protein extraction was performed first. After removal of the ethanol, 40 µL of 70% (vol/vol) formic acid (Merck) was added and heavily mixed to lyse the cells. Then, 40 µL of acetonitrile (Merck) was added to suspend the proteins. This mixture was microcentrifuged (21,100 × *g*, 3 min, 4°C). Afterward, 1 µL of the upper layer, the protein extract, was spotted on a target plate (MSP 96 target polished steel plate; Bruker Daltonics, Billerica, Massachusetts, USA) and air dried. Subsequently, 1 µL of matrix solution was added [10 mg of α-cyano-4-hydroxycinnamic acid (Merck) dissolved in 1 mL of solvent (acetonitrile:water:trifluoroacetic acid, 50:47.5:2.5)]. The target plate was then placed into a MicroflexTM LT/SH smart system (Bruker Daltonics) for analysis. After the mass spectra were obtained, they were compared with those of a commercially available database (BDAL MSP-9445; Bruker Daltonics) and an UGent in-house available database (MSP-2862) by means of the software Compass Explorer (Bruker Daltonics). Then, the mass spectra were automatically dereplicated with the data analysis tool SPeDE, which runs with Python software (84). A second dereplication was done with the BioNumerics software (version 7.6.3; Applied Maths, Sint-Martens-Latem, Belgium) using Pearson correlation and the unweighted pairwise group method with mathematical averages.

#### Confirmation of the identification by gene sequencing

Identification of the MALDI-TOF MS clusters was confirmed by sequencing of the most suitable genetic regions of representative isolates of each cluster. Therefore, cultures of the selected isolates were first subjected to a genomic DNA extraction, following the protocol of the Nucleospin 96 Tissue Kit (Macherey-Nagel, Düren, Germany), as described previously (85). First, an enzymatic lysis with lysozyme (120,000 U; Merck) and mutanolysine (100 U; Sigma-Aldrich) for bacterial isolates and with lyticase (200 U; Sigma-Aldrich) and Zymolyase (1.5 U; G-Biosciences, St. Louis, Missouri, USA) for fungal isolates was performed. After removal of the proteins with 25 µL of proteinase K (28.85 g/L; Macherey-Nagel), the DNA was, after addition of lysis buffer BQ1, transferred to the silica membranes of the Nucleospin binding plate, which was washed with washing buffers BW and B5, and eluted in elution buffer BE, all supplied by Macherey-Nagel and used as described in the manufacturer’s protocol.

For fungi, the internal transcribed spacer (ITS) region (ITS1-ITS4) was targeted. For LAB, the full-length 16S rRNA gene was used. For AAB, both the 16S rRNA gene and *dna*K gene were sequenced. For the amplification of the bacterial 16S rRNA gene through a polymerase chain reaction (PCR) assay, the primers pA (5′-AGAGTTTGATCCTGGCTCAG-3′) and pH (5′-AAGGAGGTGATCCAGCCGCA-3′) were used (86), in a mixture consisting of 36.75 µL of MilliQ, 5 µL of 10× PCR buffer (Roche Diagnostics, Basel, Switzerland), 2.5 µL of bovine serum albumin (BSA, 0.1 mg/mL; Acros Organics, Geel, Belgium), 2 µL of each of the primer solutions (5 µM; Integrated DNA Technologies, Leuven, Belgium), 0.5 µL of a deoxynucleotide triphosphate (dNTP) solution (5 mM of each of the dNTPs; Sigma-Aldrich), 0.25 µL of *Taq* DNA polymerase (5 U/µL; Roche Diagnostics), and 1 µL of the bacterial DNA extracted. The program followed for the amplification consisted of a denaturation at 95°C for 4 min, followed by three cycles of denaturation (95°C for 45 s), annealing (55°C for 2 min), and extension (72°C for 1 min) and 30 cycles of denaturation (95°C for 20 s), annealing (55°C for 1 min), and extension (72°C for 1 min), followed by a final extension at 72°C for 7 min. The *dna*K gene was amplified using the primers dnaK-01-F (5′-CTGCGCATCATCAACGAGCC-3′) and dnaK-02-R (5′-CTCACGCTCGCCCTGATAGA-3′) (87), which were used in the PCR assay mixture described above. The thermal program started with a denaturation at 95°C for 5 min, followed by two cycles of denaturation at 95°C for 60 s, annealing at 55°C for 135 s, and extension at 72°C for 75 s and then 29 cycles of denaturation at 95°C for 35 s, annealing at 55°C for 75 s, and extension at 72°C for 75 s, followed by a final extension at 72°C for 7 min. For amplification of the fungal ITS region, the primers ITS1 (5′-TCCGTAGGTGAACCTGCGG-3′) and ITS4 (5′-TCCTCCGCTTATTGATATGC-3′) were used (88), which were added to the PCR assay mixture described above. The PCR program consisted of an initial denaturation at 95°C for 5 min, followed by 34 cycles of denaturation at 95°C for 30 s, annealing at 52°C for 1 min, and extension at 72°C for 2 min and a final extension at 72°C for 7 min.

The PCR products were purified with the Wizard SV Gel and PCR Clean-up system (Promega, Madison, Wisconsin, USA), according to the manufacturer’s instructions, to remove DNA fragments smaller than 100 bp. Afterward, these purified PCR products were sequenced through Sanger sequencing in a commercial facility (Macrogen, Amsterdam, The Netherlands) (89). After sequencing, the forward and reverse fragments were aligned to each other, making use of the BioEdit-Software (version 7.2) (90). The resulting consensus sequences were compared with the GenBank database of the National Center for Biotechnology Information (NCBI, Bethesda, Maryland, USA; https://blast.ncbi.nlm.nih.gov/), making use of the basic local alignment search tool algorithm to determine the closest type strains (91). Isolates for which the consensus sequence yielded a nucleotide identity ≥ 98.0% with the type strains were considered tentatively identified at the species level, and the accession numbers of the closest related type strains are given.

### Culture-independent identification

#### Total DNA extraction

The cell pellets obtained after centrifugation of the beer samples (see the “Sampling” section above) were subjected to a total DNA extraction to assess the microbial diversity of these samples in a culture-independent way by high-throughput sequencing of amplicon-based fragments. Therefore, a DNA extraction protocol that consisted of an enzymatic lysis, chemical treatment, and physical disruption was followed, as described previously, with some minor changes (92). To lyse the fungal cells, the cell pellets were resuspended in 600 µL of sorbitol buffer [1.2 M sorbitol (VWR International), 50 mM Tris-base (Merck), pH of 7.5], containing 30 mM β-mercaptoethanol (Merck), 15 U of Longlife Zymolyase (G-Biosciences), and 200 U of lyticase (Sigma-Aldrich), and this mixture was incubated, with regular shaking, at 30°C for 1 h. Next, the samples were microcentrifuged (6,000 × *g*, 10 min) and the supernatant was discarded. A second enzymatic lysis step, to lyse the bacterial cells, was performed by treating the cell pellets with 20 mg/mL of lysozyme (Merck) and 100 U of mutanolysin (Sigma-Aldrich) in 400 µL of STET buffer [234 mM sucrose (Merck), 50 mM ethylenediaminetetraacetic acid (EDTA; Sigma-Aldrich), 50 mM Tris-base (Merck), and 201 mM Triton X-100 (Sigma-Aldrich)]. After an incubation of 1 h at 37°C, 40 µL of 20% (m/vol) of sodium dodecyl sulphate (SDS; Sigma-Aldrich) and 0.2 g of acid-washed glass beads (Sigma-Aldrich) were added to the mixtures, which were mixed for 60 s, followed by the addition of 50 µL of proteinase K (2 mg/mL; Merck) before incubation at 56°C for 1 h to hydrolyze the proteins. Then, 500 µL of chloroform:phenol:isoamyl alcohol [49.5:49.5:1.0 (vol/vol/vol); Sigma-Aldrich] was added, and after 5 min of mixing, the mixture was transferred to phase lock tubes (5PRIME; Quantabio, Beverly, Massachusetts, USA). These mixtures were microcentrifuged (18,000 × *g*, 5 min), the upper layer was transferred to new microtubes, to which 11 µL of RNase (Roche Diagnostics) was added, and these mixtures were incubated at 37°C for 10 min. From this point, the DNA was purified with the DNeasy Blood and Tissue Kit (Qiagen, Venlo, The Netherlands) according to the manufacturer’s instructions. Final elution of the DNA from the Dneasy Mini Spin columns was performed with 100 µL of nuclease-free water (VWR International). Finally, a NanoDrop ND-2000 spectrophotometer (Thermo Scientific) was used to measure the DNA concentrations.

#### Amplification and high-throughput sequencing of marker genes

To assess the bacterial communities in the beer samples, the full-length 16S rRNA gene was amplified, making use of the primers 27F (5′-AGRGTTYGATYMTGGCTCAG-3′) and 1492R (5′-RGYTACCTTGTTACGACTT-3′) (93, 94). To the 5′-end of the primers, a sample-specific barcode and a spacer sequence (GCATC) were added. The PCR assay mixture contained 1.5 µL of nuclease-free water (VWR), 12.5 µL of 2× KAPA HiFi DNA Polymerase (Hot Start and Ready Mix formulation; Roche Diagnostics), 3 µL of each primer (2.5 µM; Integrated DNA Technologies), and 5 µL of the total DNA extracted (0.4 ng/µL). The PCR conditions used consisted of an initial denaturation at 95°C for 3 min, followed by 24 cycles of denaturation at 95°C for 3 min, annealing at 57°C for 30 s, and extension at 72°C for 1 min. The fungal diversity of the beer samples was assessed by amplifying the ITS1 region (95). Therefore, the primers BITS (5′-TCGTCGGCAGCGTCAGATGTGTATAAGAGACAG-3′) and B58S3 (5′-GTCTCGTGGGCTCGGAGATGTGTATAAGAGACAGGAGATCCRTTGYTRAAAGTT-3′) were used (96). The PCR assay mixture contained 36.75 µL of MilliQ, 5 µL of 10× PCR buffer (Roche Diagnostics), 2.5 µL of BSA (0.3 mg/mL; Sigma-Aldrich), 0.25 µL of dNTP mixture (2 nM each; Sigma-Aldrich), 2 µL of both primers (5 µM; Integrated DNA Technologies), 0.25 µL of *Taq* DNA polymerase (5 U/µL; Roche Diagnostics), and 1 µL of the total DNA extracted (50 ng/µL). The PCR conditions used consisted of an initial denaturation at 95°C for 2 min, followed by 40 cycles of denaturation at 95°C for 30 s, annealing at 55°C for 30 s, and extension at 72°C for 1 min, followed by a final extension at 72°C for 5 min. After amplification, a purification of both the bacterial and fungal PCR products was performed with the Wizard Genomic DNA Purification Kit (Promega) according to the manufacturer’s instructions.

For the bacterial amplicons, the concentrations were measured with a Qubit 2.0 fluorometer (Thermo Scientific) and the length distribution was checked with a Bioanalyzer 2100 (Agilent Technologies, Santa Clara, California, USA). Next, the samples were pooled in an equimolar ratio, circular sequencing adaptors were ligated to the amplicons, and the resulting library was sequenced with a Pacific Biosciences Sequel system (Pacific Biosciences, Menlo Park, California, USA) in circular consensus mode in a sequence facility (VIB Nucleomics Core Facility, Leuven, Belgium). The sequence reads obtained were clustered with the DADA2 package for the R software (version 1.14.1) to obtain ASVs (93). The following filtering parameters were used in the DADA2 pipeline: MinQ = 3, MaxEE = 2, Minlength = 1100, and Maxlength = 1600. Finally, the taxonomy of the ASVs was assigned using the SILVA database (version 138) (97), setting the minboots parameter at 80 as cut-off. The ASVs of the three separate beer samples were taken together to determine their relative abundances at each maturation time point.

Fungal amplicons were, after purification, subjected to a size selection. This was done as described previously (95), with minor modifications. Here, the volume of magnetic beads added to the samples was 1.2 times the volume of the samples, to select for shorter amplicons because of the highly variable length of the fungal ITS1 region. The concentrations were measured with a Qubit 2.0 system (Thermo Scientific), and the quality was checked with a Bioanalyzer 2100 (Agilent Technologies). The sequencing was performed with an Illumina MiSeq platform (Illumina, San Diego, California, USA) in a sequencing facility (BRIGHTcore, Jette, Belgium). The sequence reads obtained were clustered with the DADA2 software package to obtain ASVs (98, 99) with the trimming parameters truncQ = 2, MaxEE = 1, and Minlength = 50, and their taxonomy was assigned using the UNITE database (version 8, 02.02.2019) (100), setting the minboots parameter at 50 as cut-off. The ASVs of the three separate beer samples were taken together to determine their relative abundances at each maturation time point.

### Metabolite target analysis

The substrates and microbial metabolites targeted were all measured in the cell-free supernatants obtained after thawing of the samples stored (see the “Sampling" section above) by combining several chromatographic separation methods with the most appropriate detection techniques. All samples were measured in triplicate and were quantified with external calibration.

#### Determination of carbohydrate concentrations

The concentrations of simple saccharides, i.e*.*, glucose, fructose, sucrose, and maltose, and maltooligosaccharides, i.e*.*, maltotriose, maltotetraose, maltopentaose, maltohexaose, maltoheptaose, and maltooctaose, were quantified by high-performance anion exchange chromatography coupled with pulsed amperometric detection (HPAEC-PAD), as described previously (36, 37, 101). Therefore, an ICS3000 chromatograph (Dionex, Sunnyvale, California, USA), which was equipped with a Carbopac PA20-column (for simple carbohydrates) and a Carbopac PA100-column (for maltooligosaccharides), coupled to an ED-40 PAD-detector (Dionex) was used. To prepare the samples, the beer samples were first diluted, if necessary, and then, 100 µL of this (diluted) beer sample was mixed with 900 µL of a deproteinization solution [50% (vol/vol) MilliQ, 50% (vol/vol) acetonitrile (Fisher Scientific), and 15 mg/L of rhamnose (Fluka Chemie, Buchs, Switzerland) as internal standard (IS)]. After mixing, the samples were microcentrifuged (21,100 × *g*, 15 min, 4°C) and filtered (0.2 µm Millex LG filter units; Merck Millipore; Watford, Ireland) before injection (10 µL) into the column. For the determination of the concentrations of the simple saccharides, the mobile phase consisted of three different eluents, namely, MilliQ (eluent A), 167 mM sodium hydroxide (Thermo Scientific; eluent B), and 500 mM sodium hydroxide (eluent C). The following gradient of the mobile phase (0.4 mL/min) was used: 0.0 to 15.0 min, 87.0% A, 13.0% B, and 0% C; 15.1 to 25.0 min, 0% A, 0% B, and 100.0% C; and 25.1 to 35.0 min, 87.0% A, 13.0% B, and 0% C. For the determination of the maltooligosaccharide concentrations, the mobile phase consisted of 100 mM sodium hydroxide (eluent A), 1,000 mM sodium hydroxide (eluent B), and 100 mM sodium hydroxide with 380 mM sodium acetate (eluent C). The following gradient was used at 1.0 mL/min: 0.0 to 5.0 min, 96.0% A, 4.0% B, and 0% C; 5.0 to 15.0 min, linear from 96.0 to 60.0% A and from 4.0 to 40.0% B; 15.0 to 20.0 min, linear from 60.0 to 30.0% A and from 40.0 to 70.0% B; 20.1 to 25.0 min, 100.0% B; 25.1 to 33.0 min, 0% A, 0% B, and 100.0% C; and 33.1 to 40.0 min, 0% A, 96.0% B, and 4.0% C.

#### Determination of organic acid concentrations

The concentrations of lactic acid and acetic acid were determined by high-performance liquid chromatography with photodiode array detection (PDA) and refractive index (RI) detection, respectively, as described previously (36, 37). Therefore, a Waters chromatograph (Waters, Milford, Massachusetts, USA) was equipped with an ICSep ICE ORH-801 column (Transgenomic North America, Omaha, Nebraska, USA) and W2996 PDA and W410 RI detectors (both from Waters). The samples were prepared by adding 300 µL of Carrez A solution [36 g/L of potassium hexacyanoferrate trihydrate (Sigma-Aldrich)] and 300 µL of Carrez B solution [72 g/L of zinc sulphate heptahydrate (VWR)] to 600 µL of beer sample. After mixing and microcentrifugation (21,100 × *g*, 15 min, 4°C), the supernatant was filtered (0.2 µm Millex LG filter units; Merck Millipore). Then, 30 µL of the sample was injected into the column (35°C). The mobile phase consisted of 5 mM sulfuric acid (Merck) and was isocratic at 0.4 mL/min.

The concentrations of citric acid, malic acid, and succinic acid were determined by ultra-performance liquid chromatography with tandem mass spectrometry detection (UPLC-MS/MS), as described previously (102). An Acquity UPLC system with a HSS T3 column and a TQ tandem mass spectrometer, all from Waters, were used. The samples were prepared by mixing 40 µL of beer sample with 760 µL of a deproteinization solution [50% (vol/vol) MilliQ, 50% (vol/vol) methanol (Fischer Scientific), and 20 ppm of salicylic acid (Fluka Chemie) as IS]. Then, the samples were microcentrifuged (21,100 × *g*, 15 min, 4°C) and filtered (0.2 µm Millex LG filter units; Merck Millipore). Next, 2 µL of sample was injected into the column. Two eluents, eluent A [98.0% (vol/vol) MilliQ, 2.0% (vol/vol) methanol, and 53 mM formic acid (Merck)] and eluent B [95.0% (vol/vol) methanol, 5.0% (vol/vol) MilliQ, and 53 mM formic acid], were used to compose the mobile phase. The following gradients were used at a flow rate of 0.23 mL/min: from 0.0 to 1.5 min, 92.0% eluent A and 8.0% eluent B; from 1.5 to 3.0 min, a linear decrease of eluent A to 10.2% and a linear increase of eluent B to 89.8%; from 3.0 to 6.0 min, 10.2% eluent A and 89.8% eluent B; from 6.0 to 6.5 min, a linear increase of eluent A to 92.0% and a linear decrease of eluent B to 8.0%; and from 6.5 minto 10.0 min, 92.0% eluent A and 8.0% eluent B.

#### Determination of volatile organic compound concentrations

First, a fingerprint of the VOCs present in the beer samples was composed through HS/SPME-GC-TOF-MS (102). Therefore, 1 mL of beer sample was transferred to glass vials of 5 mL, after which the fiber (divinylbenzene/carboxen/polydimethylsiloxane, 50/30 µm; Merck) was inserted into the headspace above the liquid phase. After 5 min of incubation at 45°C, the fiber was transferred to the iConnect helium saver split/splitless injector (Thermo Scientific), which was at a temperature of 250°C. In the injector, a split flow (with nitrogen gas; Nippon Gasses, Antwerp, Belgium) of 20.0 mL/min and a carrier flow (helium gas; Nippon Gasses) of 1 mL/min were set, resulting in a split ratio of 20. The gas chromatograph, a Trace1310 system (Thermo Scientific), was equipped with a DBWax-UI column (Agilent, Santa Clara, California, USA) and coupled to a BenchTOF mass spectrometer (Markes International, Llantrisant, Wales, UK). The following oven program was used: 0.0 to 1.5 min, constant at 40°C, from 1.5 to 20 min, linear increase from 40°C to 225°C at a rate of 10°C/min, and from 20.0 to 35.0 min, constant at 225°C. The BenchTOF mass spectrometer operated in positive electron ionization mode, scanning a *m/z* range from 36 to 400. The ionization temperature was set at 250°C. After the mass spectra were retrieved, they were compared with the NIST14 library (National Institute of Standards and Technology, Gaithersburg, Maryland, USA). The samples were run in triplicate, and VOCs that were detected in two of the three replicates were selected for further quantification.

For the quantification of the concentrations of several alcohols, i.e*.*, ethanol, 2/3-methyl-1-butanol, 2-methyl-1-propanol, and 2-phenylethanol, and of the most abundant ethyl esters, i.e*.*, ethyl lactate and ethyl acetate, gas chromatography with flame ionization detection was used, as described previously, with some adaptations (36, 37). A Trace1310 gas chromatograph (Thermo Scientific) was equipped with a Stabilwax-DA column (Restek, Bellefonte, Pennsylvania, USA) and coupled to an iConnect FID detector (Thermo Scientific). The samples were prepared by adding 900 µL of a deproteinization mixture [75% (vol/vol) acetonitrile (Thermo Scientific), 25% (vol/vol) MilliQ, 318.1 mM formic acid (Merck), and 2.7 mM 1-butanol (Sigma-Aldrich) as IS]. After mixing, the samples were centrifuged (21,100 × *g*, 15 min, 4°C) and filtered (0.2 µm Millex LG filter units; Merck Millipore). Then, 0.8 µL of sample was injected into the inlet, which had an initial temperature of 250°C. The split flow and carrier flow, both helium (Nippon Gasses), were set at 40 and 1 mL/min, respectively, resulting in a split ratio of 40. The temperature of the column oven was kept constant at 60°C for 1.5 min, followed by a linear increase between 1.5 and 19.5 min from 60°C to 240°C at a rate of 10°C/min, and a constant temperature of 240°C between 19.5 and 30.0 min. For the detector, nitrogen gas (Nippon Gasses) was used as make-up gas, while hydrogen gas [generated by an H2PD hydrogen generator (Parker Hannifin, Cleveland, Ohio, USA)] and oxygen gas (compressed air) were used for the detector.

Other VOCs, such as other esters, volatile phenolic compounds, and metabolites related to the pyruvate metabolism, were quantified making use of liquid injection gas chromatography with triple-quadrupole mass spectrometry detection (LI-GC-TQ-MS/MS). Therefore, 100 µL of beer sample was mixed with a solution of acetone (Merck) containing three ISs (ethyl decanoate-d_19_, 3-methyl-1-butyl-1,1-d_2_ alcohol, and 2,3-butanedione-d_6_; all from CDN Isotopes, Pointe-Claire, Quebec, Canada). Next, the samples were centrifuged (21,100 × *g*, 15 min, 4°C) and filtered (0.2 µm Millex LG filter units; Merck Millipore) before 0.8 µL was injected into a DBwax UI column (Agilent) of a Trace1310 gas chromatograph (Thermo Scientific), coupled to a TSQ 800 Evo detector (Thermo Scientific). The iConnect helium saver split/splitless injector was set at a constant temperature of 260°C. The split flow (nitrogen gas; Nippon Gasses) was set at 5 mL/min, while the carrier gas (helium, Nippon Gasses) was set at 1 mL/min, resulting in a split ratio of 5. The following column oven program was used: from 0.0 to 1.5 min, constant at 40°C; from 1.5 to 20.0 min, linear increase at 10°C/min; from 20.0 to 30.0 min, constant at 225°C; from 30.0 to 30.75 min, linear increase at 20°C/min; and from 30.75 to 40.25 min, constant at 240°C. The ion source temperature of the TSQ was set at 280°C, and an electron ionization mode was chosen. A positive selected reaction monitoring scan was performed for a *m/z* range between 46 and 300.

#### Determination of biogenic amine concentrations

The concentrations of biogenic amines were quantified by UPLC-MS/MS (103). Therefore, the same equipment as for the determination of organic acid concentrations was used, as described above (see the “Determination of organic acid concentrations” section above). After deproteinization of 250 µL of beer sample with 750 µL of acetonitrile (Fischer Scientific), the samples were mixed and centrifuged (21,100 × *g*, 15 min), after which 100 µL of the supernatant was mixed with 400 µL of MilliQ containing 11.5 mM heptafluorobutyric acid (Alfa Aesar, Ward Hill, Massachusetts, USA). Hereafter, 2 µL was injected into the column. A constant flow rate of 0.23 mL/min was applied for the mobile phase. Eluent A consisted of 95% (vol/vol) MilliQ and 5% (vol/vol) acetonitrile (Fischer Scientific) with 7.6 mM heptafluorobutyric acid and eluent B consisted of 95% (vol/vol) acetonitrile and 5% (vol/vol) MilliQ with 7.6 mM heptafluorobutyric acid. The following gradient was applied: from 0.0 to 0.5 min, 99.0% eluent A and 1.0% eluent B; from 0.5 to 2.0 min, a linear decrease of eluent A to 90.0% and a linear increase of eluent B to 10.0%, followed by a further decrease of eluent A to 40.0% and 30.0% and an increase of eluent B to 60.0% and 70.0%, after 8.0 and 8.5 min, respectively; from 8.5. to 12.0 min, 30.0% eluent A and 70.0% eluent B; from 12.0 to 12.5 min, a linear increase of eluent A from 30.0% to 99.0 % and a decrease of eluent B from 70.0% to 1.0 %; and from 12.5 to 16.0 min, 99.0% of eluent A and 1.0% of eluent B.

### Sensory analysis

The matured beers of 13 weeks (kept at 4°C until evaluation) and 6 months were assessed by means of a triangle test by 16 untrained panel members. Three samples were served, of which one odd sample that had to be indicated. Subsequently, the presumed odd sample had to be described based on the intensity (much lower, lower, equally, higher, and much higher) of five taste descriptors, namely, sourness, fruitiness, spiciness, woodiness, and metallicness, as compared with the two other samples.

### Statistical analysis

To check whether the physicochemical parameters, microbial counts, and concentrations of various compounds significantly differed (*P <* 0.05) between the samples taken at the different time points, first, the normality was tested with a Shapiro-Wilk test and the variance was tested by performing an *F-*test. Based on the outcome of these tests, a *t*-test, Welch-corrected *t*-test, or Wilcoxon rank sum test was performed to check for significant differences between two different time points. Also, the alpha diversity of the amplicon-based sequence data at each time point was determined with the vegan package (version 2.6) (104). Therefore, the diversity (Simpson), evenness (Pielou), and true richness (Chao1) indices at each time point (sum of the three separate beer samples per time point) were calculated. All statistical analyses were performed with the statistics software R (version 4.2.3) (105). A chi-squared test was performed to determine significant differences among the beers subjected to a sensory analysis.

## Data Availability

The sequence data obtained are available under BioProject PRJEB71653. The PacBio amplicon sequences for the full-length 16S rRNA gene are available under accession numbers ERR12406583–ERR12406618, and the Illumina MiSeq amplicon sequences for the ITS region are available under accession numbers ERR12406622–ERR12406657 in the European Nucleotide Archive of the European Bioinformatics Institute (ENA/EBI).
